# A Nanosystem Capable of Releasing a Photosensitizer Bioprecursor under Two‐Photon Irradiation for Photodynamic Therapy

**DOI:** 10.1002/advs.201500254

**Published:** 2015-11-25

**Authors:** Hao Wu, Fang Zeng, Hang Zhang, Jiangsheng Xu, Jianrong Qiu, Shuizhu Wu

**Affiliations:** ^1^ College of Materials Science and EngineeringState Key Laboratory of Luminescent Materials and DevicesSouth China University of TechnologyGuangzhou510640P.R. China; ^2^ Institute of Optical Communication MaterialsState Key Laboratory of Luminescent Materials and DevicesSouth China University of TechnologyGuangzhou510640P.R. China

**Keywords:** 5‐aminolevulinic acid, mitochondrial‐targeted, photocontrolled prodrug, photodynamic therapy, two‐photon absorption

## Abstract

The applications of photodynamic therapy (PDT) are usually limited by photosensitizers' side effects and singlet oxygen's short half‐life. Herein, a mitochondria‐targeted nanosystem is demonstrated to enhance the PDT efficacy by releasing a bio‐precursor of photosensitizer under two‐photon irradiation. A phototriggerable coumarin derivative is first synthesized by linking 5‐aminolevulinic acid (5‐ALA, the bio‐precursor) to coumarin; and the nanosystem (CD‐ALA‐TPP) is then fabricated by covalently incorporating this coumarin derivative and a mitochondria‐targeting compound triphenylphosphonium (TPP) onto carbon dots (CDs). Upon cellular internalization, the nanosystem preferentially accumulates in mitochondria; and under one‐ or two‐photon irradiation, it releases 5‐ALA molecules that are then metabolized into protoporphyrin IX in mitochondria through a series of biosynthesis processes. The subsequent red light irradiation induces this endogenously synthesized photosensitizer to generate singlet oxygen, thereby causing oxidant damage to mitochondria and then the apoptosis of the cells. Analysis via 3‐(4,5‐dimethyl‐2‐thiazolyl)‐2,5‐diphenyltetrazolium bromide (MTT) assays indicate that the novel PDT system exhibits enhanced cytotoxicity toward cancer cells. This study may offer a new strategy for designing PDT systems with high efficacy and low side effects.

## 1 Introduction

In the last few decades, photodynamic therapy (PDT) has garnered enormous interests as a safe, minimally invasive treatment for numerous cancers and certain noncancerous diseases.^1^ PDT is a treatment method combining light, photosensitizer, and molecular oxygen. Photosensitizer can produce reactive oxygen species (ROS) under light irradiation, such as singlet oxygen (^1^O_2_), superoxide anion radical (O^2−^), and hydroxyl radical (^−^OH), and relies on the cytotoxic effects of ROS to lead to apoptosis or necrosis of abnormal cells.^2^ As a class of common photosensitizers, porphyrins were studied extensively in clinical PDT during the past two decades and have been approved by many countries for several medical indications.^3^ However, some side effects associated with porphyrin‐based PDT do exist, including the risk of skin photosensitization and damages to surrounding normal tissues potentially leading to stenosis of the esophagus and shrinkage of the bladder.^4^ And these side effects restrict the use of porphyrin‐based PDT.

Alternatively, 5‐aminolevulinic acid (5‐ALA), a bio‐precursor of protoporphyrin IX (PPIX), has gained considerable attentions in recent years, and PDT with ALA‐derived endogenous porphyrins has already shown promising clinical results in the treatment of several superficial disorders of the skin (such as actinic keratosis, a skin condition that will further develop into cancer) and internal hollow organs.^5^ 5‐ALA is an endogenous amino acid, which is usually synthesized from glycine and succinyl CoA in mitochondria. Induced by supplementation of 5‐ALA in mitochondrion, endogenous porphyrins biosynthesis occurs through several enzyme‐catalyzed steps in cytosol and mitochondrion.^6^ On the other hand, following systemic administration, exogenous 5‐ALA can also be metabolized into PPIX via the same biosynthesis pathway.^7^ Unlike some other photosensitizers, 5‐ALA may function in cancer treatment with less risk of phototoxicity as an endogenous product, since it will not cause damage to normal tissues before internalization by cancer cells. However, the use of 5‐ALA in PDT is limited by the low cell penetration capacity of this highly hydrophilic molecule as well as its low stability in biological fluids.[[qv: 7a]],^8^ Thus, to facilitate the bioavailability of 5‐ALA, an improved means of delivery is highly desirable to convey 5‐ALA to the specific sites and then release it efficiently.

Currently, there are several means available to serve as the external stimuli to trigger the release of drugs from their vehicles;^9^ and light is a particularly advantageous one. It can function from outside the diseased site and provide spatiotemporal control of payload release with great ease and convenience. So far, a number of light‐responsive compounds have been explored and successfully used in many drug‐release systems.^10^ Among these compounds, coumarin family has been widely utilized in photo‐cleavable applications.^11^ Coumarin and its derivatives are well known to undergo reversible dimerization and photocleavage of cyclobutane under light irradiation; and more importantly, this photocleavage can be activated by two‐photon near‐infrared (NIR) irradiation.^12^ Compared to the short wavelength light, NIR can penetrate much deeper (up to a centimeter) into tissues with less photo‐induced damages.^13^

**Scheme 1:**
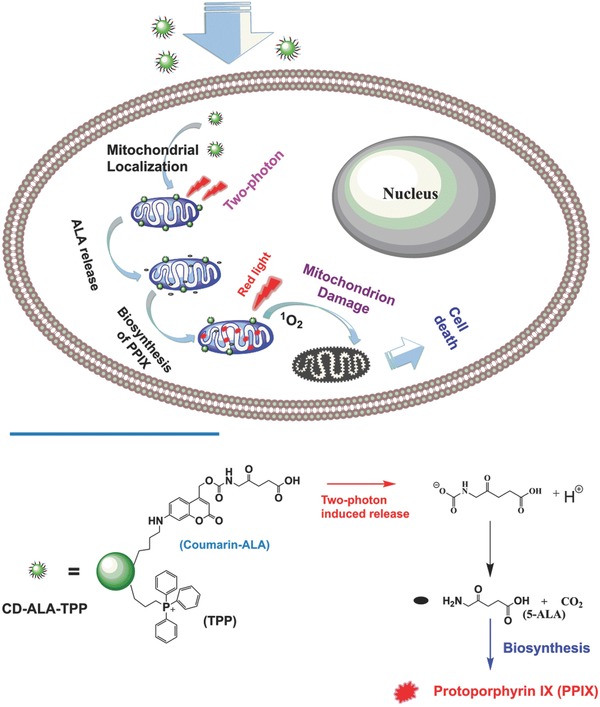
Schematic illustration for the photo‐triggered release of ALA from CD‐ALA‐TPP and the subsequent proapoptotic action on a cancer cell. The numbers of coumarin‐ALA and TPP on the carbon dot do not reflect the actual numbers.

We envision that, if 5‐ALA as a bioprecursor for photosensitizer can be integrated into a nanoparticle system and subsequently released intracellularly upon light illumination, then the in situ biosynthesis of photensitizer inside cells can be realized, which could greatly improve the bioavailability of 5‐ALA and in the meantime reduce the side effects associated with common PDT procedures. Herein, as a proof of concept, we for the first time formed a photo‐triggerable bioprecursor unit by covalently linking 5‐ALA (bioprecursor) to a photo‐cleavable coumarin (light trigger), and then incorporated this unit onto a nanoparticle scaffold (carbon dot, CD), thereby establishing a nanosystem for photo‐triggered release of bioprecursor inside cells. This nanoparticle‐based ALA‐releasing strategy for enhancing the efficacy of PDT is shown in **Scheme** **1**. For this strategy, CD, which features low cytotoxicity, high fluorescent emission, and good water dispersibility,^14^ serves as the fluorescent carrier for the nanosystem; and the PPIX precursor 5‐ALA is linked onto CD via a photo‐cleavable coumarin structure. Moreover, a biocompatible and hydrophobic cation triphenylphosphonium (TPP) is covalently incorporated onto the carrier to facilitate the cell penetration capability and allow for the delivery of 5‐ATA to the close vicinity of mitochondria. Upon internalization by cancer cells, the nanosystem can release 5‐ALA under violet light (one‐photon process) or NIR light (two‐photon process) irradiation, and the released payload undergoes a series of biosynthesis steps to generate PPIX in mitochondria of the cells. Upon exposure to red light, the generated PPIX effectively destroy the mitochondria of the cancer cells and result in cell apoptosis.^15^ Our results indicate that the mitochondria‐targeting and the photo‐induced release of 5‐ALA can realize more efficient PDT action toward cancer cells. The significance of this work lies in the possibility of developing a new PDT strategy which can not only avoid the common side effects of porphyrin‐based PDT (such as risks of skin photosensitization and damages to surrounding normal tissues), but also enhance the bioavailability of ALA. This targeted and two‐photon activatable delivery system may potentially be exploited in PDT‐related applications.

## 2 Results and Discussion

### 2.1 Preparation and Characterization of the Nanosystem

**Scheme 2:**
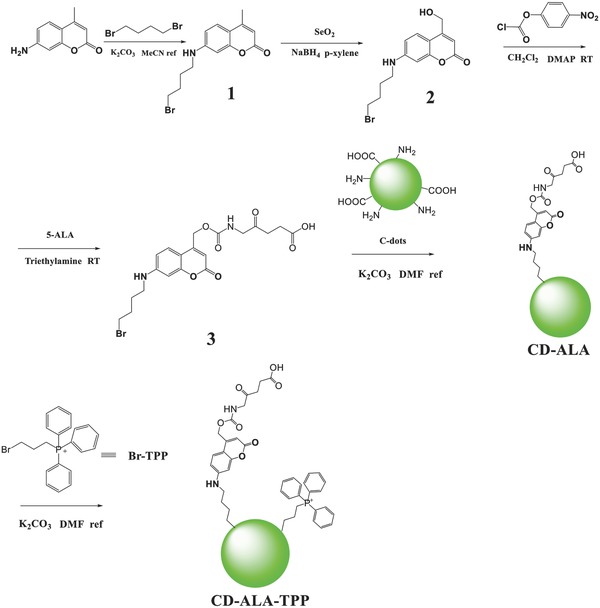
Synthesis route for CD‐ALA‐TPP and CD‐ALA. The numbers for the functional groups on the C‐dots do not reflect actual numbers. For the synthesis of the C‐dots, citric acid was used as the carbon source and urea as the surface passivation agent.

In this study, a CD‐based nanosystem CD‐ALA‐TPP was fabricated by covalently linking a 5‐ALA releasing element and a hydrophobic cation (TPP) onto green‐emitting CDs; and the procedure for the synthesis of CD‐ALA‐TPP is shown in **Scheme** **2**. For the synthesis of 5‐ALA releasing element, first an amino‐containing coumarin was modified with 1,4‐dibromobutane to form a bromo‐containing coumarin (compound **1**), and a 4‐hydroxymethyl coumarin (compound **2**) was then synthesized from compound **1** through oxidation by SeO_2_ and subsequent reduction by NaBH_4_. Afterward, 5‐ALA was linked to compound **2** via a photocleavable carbamate bond to form the ALA‐releasing element (compound **3**). To obtain CD‐ALA‐TPP, compound **3** and a bromo‐containing TPP (Br‐TPP) were all linked to CD through the reaction between bromo groups (in Br‐TPP and compound **3**) and the amino groups (on CD). Moreover, a nanosystem (CD‐ALA) which contains only compound **3** was also prepared as the control.

The ^1^H NMR and ESI‐MS spectra for compounds **1**, **2**, and **3** are given in Figures S1–S3 (Supporting Information), which prove the successful synthesis of these compounds. The ^1^H NMR spectra for CD‐ALA and CD‐ALA‐TPP are presented in Figures S4 and S5 (Supporting Information) respectively, in which the peaks at 8.4 ppm and 7.8 ppm are indicative of aromatic protons in ALA and TPP, respectively. Fourier transform infrared (FT‐IR) analysis for the nanosystems, as shown in Figure S6 (Supporting Information), suggests that the nanosystems retain the basic structure of the pristine CDs, as evidenced by the existence of C=N bond (at 1426 cm^−1^) and C=O bond (at 1640–1780 cm^−1^) in their FT‐IR spectra. These bonds are crucial for fluorescent emission. Moreover, X‐ray powder diffraction pattern for CD‐ALA‐TPP and CD‐ALA (Figure S7, Supporting Information) all displays a broad (diffuse) peak centered at >>5 Å, indicating the amorphous state of the CD‐based nanosystems.

**Figure 1:**
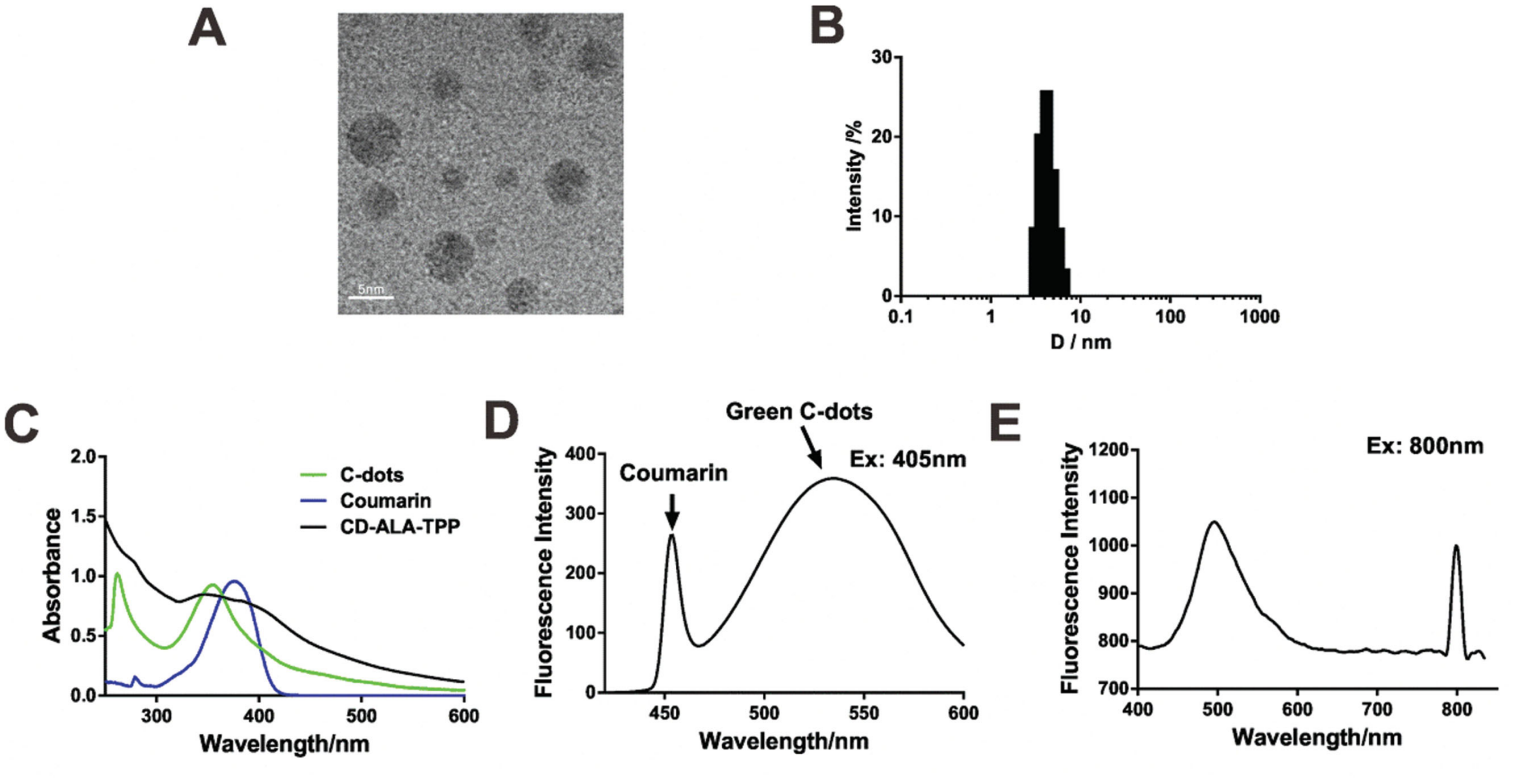
A) HR‐TEM image for CD‐ALA‐TPP. B) Particle diameter distribution as determined by dynamic light scattering DLS). C) Absorption spectrum for pristine C‐dot, CD‐ALA‐TPP, and a molecular coumarin (compound **3**). D) Emission spectrum (excited at 405 nm) for CD‐ALA‐TPP. E) Two‐photon emission spectrum for CD‐ALA‐TPP (excited at 800 nm).

TEM observation indicates that the CD‐ALA‐TPP nanoparticles generally exhibit spherical shape (**Figure** **1**A); the average diameter for the nanoparticles as determined by dynamic light scattering measurement is >>5 nm (Figure 1B). As shown in Figure 1C,D, with coumarin moieties on the C‐dot, the nanosystem exhibits an absorption band around 405 nm and an emission band at 460 nm and 550 nm (for coumarin moiety and C‐dots, respectively). The fluorescence quantum yield of the C‐dots was measured as 0.22, as shown in Figure S8 (Supporting Information). Generally, CDs exhibit quantum yield values ranging from 0.01 to 0.65, depending on the carbon source used, reaction condition, etc. The CDs prepared in this study have a moderate quantum yield among all CDs, but a relatively high value among green CDs.^14^ On the other hand, as shown in Figure 1E, under two‐photon excitation at 800 nm, the system exhibits a redshifted emission band at 500 nm. The two‐photon absorption cross section has been determined as 303.4 GM at 800 nm, as shown in the Supporting Information. Also, the photolysis (photo‐release) quantum yield for coumarin of CD‐ALA‐TPP was measured as 0.327%, as shown in the Supporting Information, and this value is moderate compared to some other coumarin‐based photolysis systems.[[qv: 11b,d]] Moreover, the amounts of TPP and ALA incorporated on the CDs have been determined as 8.34 mg g^−1^ and 24.13 mg g^−1^, respectively; and the average numbers of TPP and 5‐ALA incorporated on a single CD were subsequently estimated as 1.09 and 7.7, respectively (Figure S9, Supporting Information).

### 2.2 Release of ALA from Nanosystem upon One‐ or Two‐Photon Irradiation

**Figure 2:**
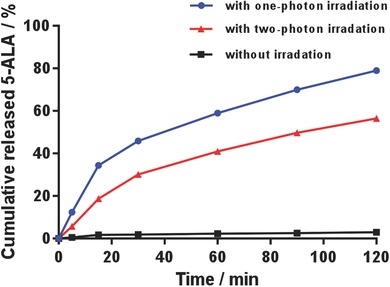
Release profile of 5‐ALA from CD‐ALA‐TPP incubated in PBS buffered water at 37 °C for varied time periods without light irradiation, with irradiation by a 10 mW cm^−2^ violet LED (wavelength ranging from 400 to 450 nm), or with irradiation by a Coherent Legend Elite Ti: sapphire regenerative amplifier system at 800 nm. The percentage of released 5‐ALA was determined using the absorption method after extraction with ethyl acetate.

The current nanosystem is designed for photo‐triggerable release of 5‐ALA inside cells under light irradiation. In this study, 5‐ALA release from the nanosystem is based on photocleavage of the C–O bond as demonstrated in Scheme 1. When excited by one or two photons, the C–O bond is cleaved, creating a carbocation that is subsequently hydrolyzed into 5‐ALA.^16^ The released ALA can react with ethyl acetoacetate to produce a pyrrole derivative, which can be extracted by ethyl acetate and then react with p‐dimethylaminobenzaldehyde to generate a red compound with a maximum absorption wavelength at 554 nm,^17^ as illustrated in Scheme S2 (Supporting Information). In this experiment, we recorded the absorption spectra (Figures S10 and S11, Supporting Information) and determined the amount of the released 5‐ALA after different time periods of light irradiation, and the results are presented in **Figure** **2**. It is clear that, without light irradiation, there is almost no 5‐ALA released from the nanosystem (Figure 2A); while under continuous irradiation, 5‐ALA can be released from the nanosystem at a rather high speed. The release profile shown in Figure 2B verifies successful activation of coumarin moieties and release of 5‐ALA by NIR light through the two‐photon process.

### 2.3 Cellular Uptake, Intracellular Localization, and ALA Release for CD‐ALA‐TPP and CD‐ALA Nanosystem

**Figure 3:**
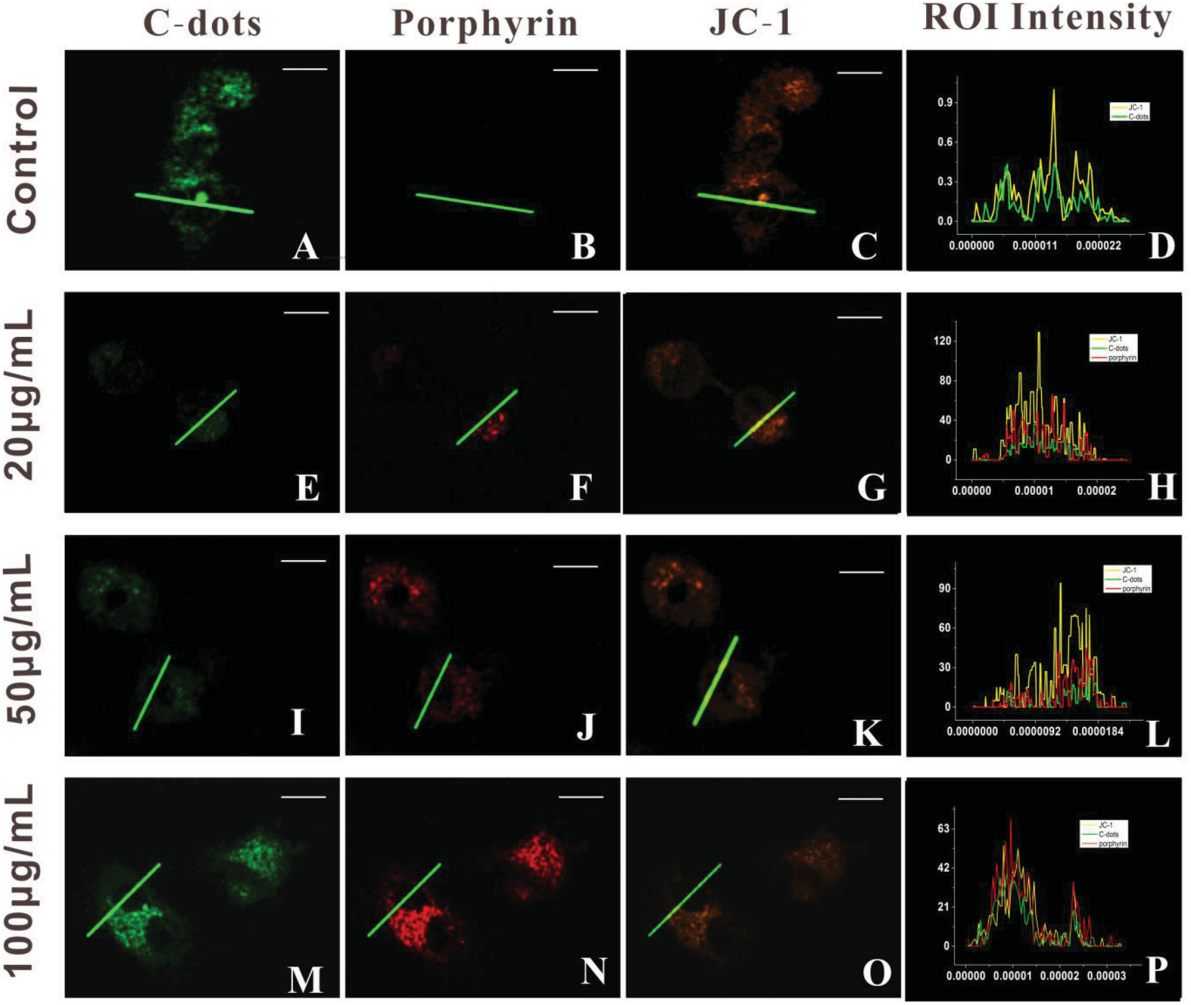
Confocal laser scanning microscopic (CLSM) images for HeLa cells co‐stained with JC‐1 and CD‐ALA‐TPP of varied concentrations followed by violet light (400–450 nm) irradiation for 30 min and red light irradiation for 15 min. A cell sample co‐stained with JC‐1 and 100 μg mL^−1^ CD‐ALA‐TPP but without light irradiation was used as the control. For C‐dot channel, the laser excitation wavelength for C‐dot was 405 nm and the fluorescence signal was collected from 500 to 550 nm (green). Porphyrin was excited at 633 nm, and its emission signal was collected from 640 to 700 nm (red). JC‐1 was excited at 488 nm, and its emission signal was collected from 570 to 600 nm (orange). All image acquisitions and analyses were performed using Leica LAS AF software. The intensity of the laser beam and the photodetector sensitivity were kept constant in order to compare the relative fluorescence intensities among experiments. Scale bar: 20 μm. A–D) Control; E–H) treated with 20 μg/mL CD‐ALA‐TPP; I–L) treated with 50 μg/mL CD‐ALA‐TPP; M–P) treated with 100 μg/mL CD‐ALA‐TPP.

The cellular uptake and intracellular behaviors for CD‐ALA‐TPP were investigated using a confocal laser scanning microscope (CLSM), as shown in **Figure** **3** (and the fluorescence microscope as shown in Figure S12, Supporting Information). Before imaging, HeLa cells were co‐stained with JC‐1 dye (a mitochondrion specific dye) and CD‐ALA‐TPP of varied concentrations, and then subject to 30 min of 400–450 nm (violet light) light irradiation so as to trigger the release of ALA. Moreover, a cell sample which was co‐stained with JC‐1 and 100 μg mL^−1^ of nanosystem but not exposed to light irradiation was used as a control.

As we can see in Figure 3, without exposure to violet light irradiation, the control sample exhibits the fluorescence of CDs (green) and that of the JC‐1 dye (orange), but the intracellular fluorescence of PPIX (red) cannot be observed, suggesting that endogenous PPIX cannot be generated without light irradiation. However, upon violet light irradiation for 30 min (to trigger the release of ALA), red fluorescence appears inside HeLa cells in a dose‐dependent manner, namely, the cells treated with nanosystem with higher concentrations exhibit stronger intracellular red fluorescence. These results clearly indicate that, with the hydrophobic cations (TPP) on their surfaces, the CD‐based nanosystem can be readily internalized by the cells. Moreover, the appearance of red fluorescence suggests the successful release of 5‐ALA inside cells as a result of light irradiation and the subsequent bioconversion from 5‐ALA to PPIX. To provide direct evidence for the PPIX production in cells treated with the nanosystem, the quantity of PPIX produced in HeLa cells was measured by using an ELISA kit (Human FEP) specialized for human PPIX assay, and the results are given in Figure S13 (Supporting Information). The result indicates that, upon being treated with 200 μg mL^−1^ of CD‐ALA‐TPP, PPIX level was determined as 664 × 10^−9^ m per 10^5^ cells. For comparison, we also tried to determine the PPIX level with the fluorescence method by recording the fluorescent intensity at 630 nm, which gave a value of 724 × 10^−9^ m per 10^5^ cells, as shown in Figure S14 (Supporting Information). We could not detect PPIX in HeLa cells without being treated with the nanosystem. The slightly higher value obtained from the fluorescence method, we suppose, may result from the contribution by some other endogenous fluorescent substances in cells.

Previous researches revealed that, the biosynthesis of PPIX from 5‐ALA is a part of heme synthesis, during which 5‐ALA is synthesized in mitochondrion and then transformed into PPIX via a complicated biosynthesis process in cytosol and in mitochondrion.^18^ In this study, we incorporated a hydrophobic cation (TPP) onto CDs, so that the nanosystem can target the mitochondria of the cells and deliver 5‐ALA in the close vicinity of mitochondria, and eventually benefit the transformation of 5‐ALA into PPIX. To confirm whether the CD‐ALA‐TPP can target and specifically stain the mitochondria, a mitochondria‐specific dye JC‐1, was employed to co‐stain HeLa cells with CD‐ALA‐TPP, and confocal images for the JC‐1 channel are presented in Figure 3C,G,K,O. By comparing the location of the observed red fluorescence from PPIX, the green fluorescence from the CDs, and the orange fluorescence from the aggregated state of JC‐1, one can find there is a remarkable overlap among them; and with the increasing concentration of nanosystem, the overlap becomes greater. The overlap is also supported by the intensity profiles of linear regions of interest given in Figure 3D,H,L,P. These results suggest that with the targeting ligand on its surface, CD‐ALA‐TPP stains specifically the mitochondrial region in live cells. For comparison, the cellular uptake for both CD‐ALA‐TPP and a control system with no TPP in its structure (CD‐ALA) was observed on a fluorescence microscope under the same experimental condition, and the result is given in Figure S15 (Supporting Information). It is clear that the cells treated with CD‐ALA‐TPP exhibit brighter intracellular fluorescence.

### 2.4 One‐ and Two‐Photon Excited Fluorescence Images for Coumarin Moieties and Imaging of Reactive Oxygen Species Generated in Live Cells

**Figure 4:**
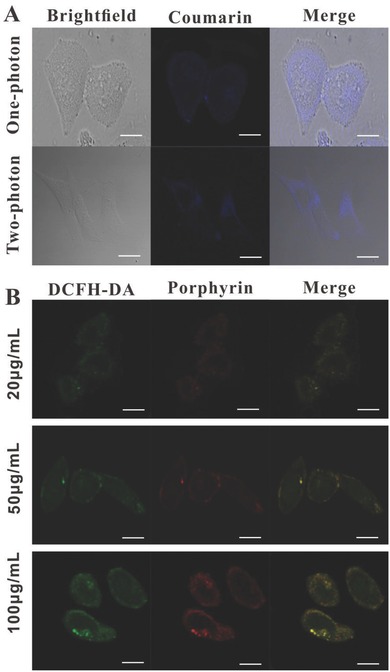
A) One‐photon (405 nm) and two‐photon (800 nm) excited CLSM images for HeLa cells treated with CD‐ALA‐TPP. The fluorescent signals were collected from 450 to 500 nm. B) CLSM images for HeLa cells treated with CD‐ALA‐TPP for 4 h followed by violet light (wavelength ranging from 400 to 450 nm, with the power of 10 mW cm^−2^) irradiation for 30 min and red light (wavelength ranging from 645 to 655 nm, 10 mW cm^−2^) irradiation for 15 min. The laser excitation wavelength for DCFH‐DA was 488 nm and the fluorescent signal collection wavelength was from 530 to 570 nm (green). PPIX was excited at 633 nm, and its emission was collected from 640 to 700 nm (red). All image acquisitions and analyses were performed using Leica LAS AF software. The intensity of the laser beam and the photodetector sensitivity were kept constant in order to compare the relative fluorescence intensities among experiments. Scale bar: 20 μm.

**Figure** **4**A shows the one‐ and two‐photon excited CLSM images for HeLa cells treated with the nanosystem. Under either one‐ (excited at 405 nm) or two‐photon (excited at 800 nm on an LSM 710 NLO with a femtosecond Ti:Sapphire laser source) excitation, the HeLa cells all exhibit blue fluorescence, which correspond to the emission of coumarin moieties in the nanosystem. In this study, the coumarin moiety exhibits a relatively high two‐photon absorption cross‐section value (303.4 GM), and the successful two‐photon imaging of coumarin moieties in HeLa cells may provide further evidence that the coumarin moiety can undergo the two‐photon process and trigger the release of ALA thereafter.

As a biosynthesis product in cells, PPIX can generate singlet oxygen with relatively high quantum yield of 0.54 when excited at 630 nm,[[qv: 15b]] to prove the generation of ROS (singlet oxygen is one kind of ROS) in the nanosystem‐treated cells upon light irradiation, we utilized a fluorescent ROS indicator 2′,7′‐dichlorodihydrofluorescein diacetate (DCFH‐DA) to image the generated singlet oxygen. In the presence of ROS, DCFH‐DA can be rapidly oxidized to a green fluorescent molecule (dichlorofluorescein, DCF). For the cells treated with both the nanosystem and DCFH‐DA, subject to violet light (for ALA release) and then subject to red light (for singlet oxygen generation) irradiation, prominent green fluorescence is observed, as shown in Figure 4B. And the higher amount of nanosystem loaded into the cells, the stronger the intracellular green fluorescence is. These results demonstrate that, singlet oxygen is generated in the cells loaded with the nanosystem upon light irradiation, and the nanosystem can be utilized as a precursor of PDT sensitizer.

### 2.5 PDT‐Induced Mitochondria Damage

In this study, PPIX that is produced with high concentration in mitochondria can cause mitochondrial damage upon light irradiation. The damage of mitochondria will cause the depolarization of mitochondria with a drop in the membrane potential (ΔΨ, a mark of cytochrome *c* translocation and begin the sequencing apoptosis);^19^ and we used JC‐1 dye to monitor the variation of mitochondrial membrane potential for HeLa cells treated with the nanosystem. At higher mitochondrial membrane potential, JC‐1 dyes exist as both aggregation and monomer state and fluoresce orange; while at lower ΔΨ, most JC‐1 dyes exist as monomer state and emit green. Thus, we can discriminate the cells with undamaged mitochondria from those with damaged mitochondria by recording the fluorescence change of JC‐1 using flow cytometry.

**Figure 5:**
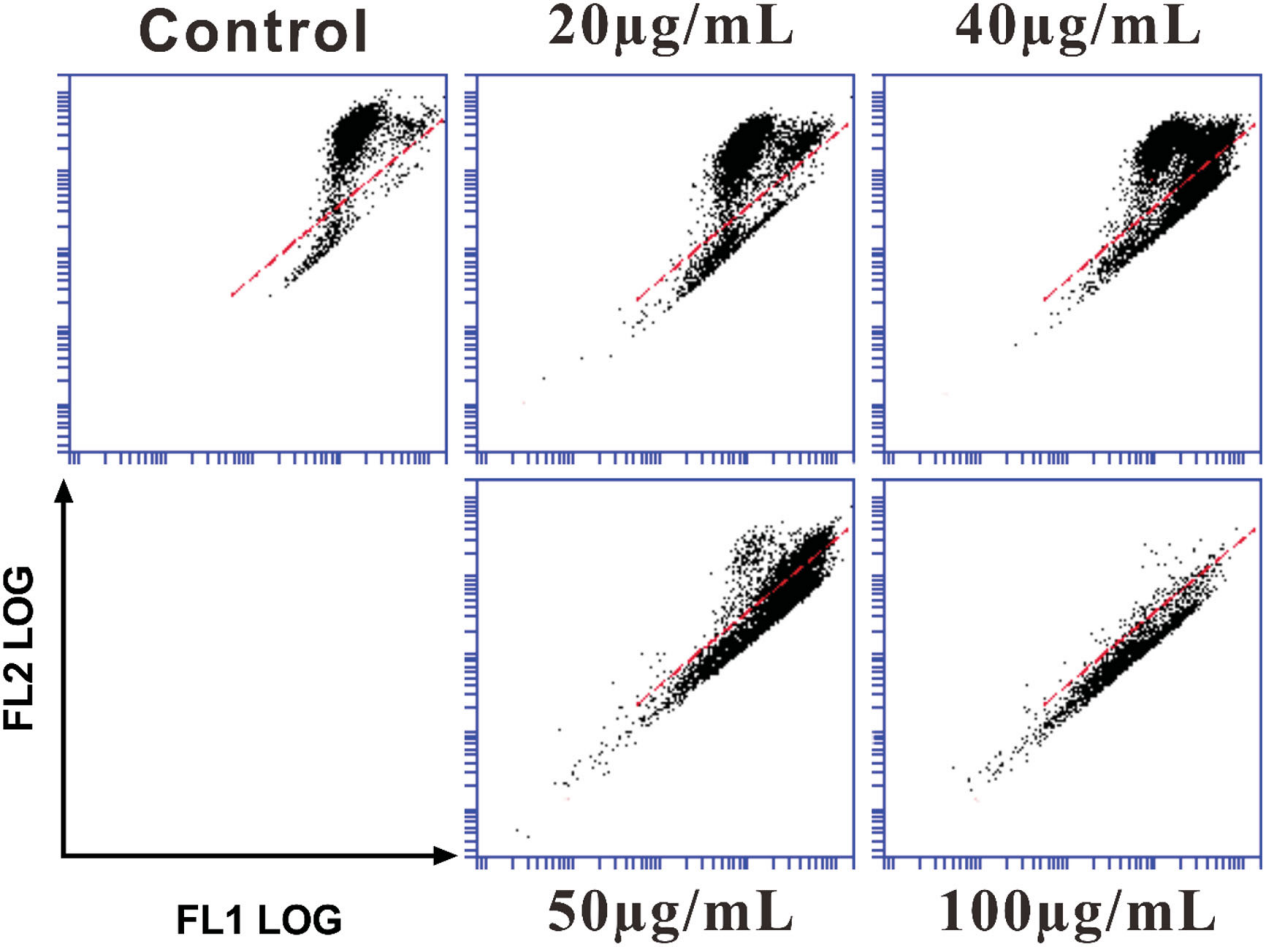
Representative flow cytometric analysis result of mitochondrial membrane potential (using JC‐1 as an indicator) for HeLa cells incubated with different concentrations of CD‐ALA‐TPP and subject to irradiation from violet LED (400–450 nm, 10 mW cm^−2^) for 30 min and red LED (645–655 nm, 10 mW cm^−2^) for 15 min. The change of JC‐1 fluorescence from orange (FL2) (570–600 nm) to green (FL1) (515–545 nm) indicates a significantly decline of mitochondrial membrane potential. Red lines separate populations with high (live cells) and low membrane potential (apoptotic cells) according to the control sample.

**Figure** **5** shows the flow cytometry profiles for HeLa cells incubated with the CD‐ALA‐TPP, irradiated with violet light and red light, and then incubated with JC‐1. A HeLa cell sample without being treated with the nanosystem is used as a control. As shown in Figure 5, without being treated by CD‐ALA‐TPP, the cells with high ΔΨ predominate in the cell population of the sample. However, following the treatment and irradiation, green fluorescence intensity (FL1 channel, for JC‐1 monomer) increases significantly over the incubation time. This result suggests that singlet oxygen generated in the mitochondria can cause mitochondria damage.

### 2.6 PDT‐Induced Apoptosis of HeLa Cells

**Figure 6:**
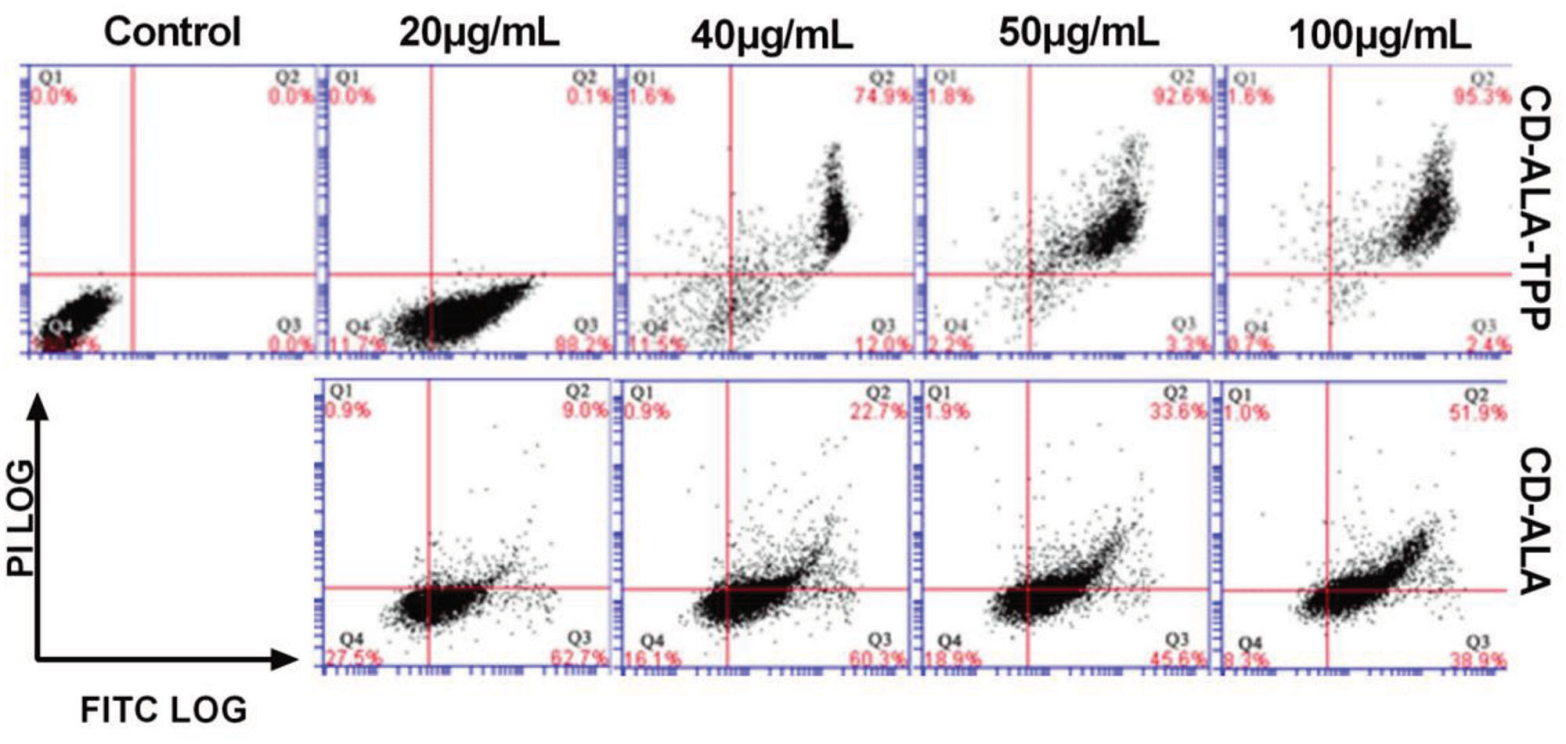
Annexin V‐FITC/Propidium Iodide (PI) dual staining for apoptosis analysis. Before the flow cytometry analysis, HeLa cells were treated with CD‐ALA‐TPP or CD‐ALA at different concentrations, respectively (control, 20 μg mL^−1^, 40 μg mL^−1^, 50 μg mL^−1^, 100 μg mL^−1^), and exposed to light irradiation from violet LED (400–450 nm, 10 mW cm^−2^) for 30 min and red LED (645–655 nm, 10 mW cm^−2^) for 15 min. Fluorescence signal for FITC was collected from FL‐1 channel (515–545 nm) and that for PI was collected from FL‐3 channel (>630 nm).

Moreover, annexin V‐FITC/PI staining assay was used to determine the proapoptosis effects of CD‐ALA‐TPP. For this purpose, HeLa cells pretreated with CD‐ALA‐TPP and V‐FITC/PI were exposed to violet light for 30 min and then red light irradiation for 15 min, followed by the flow cytometry measurement. **Figure** **6** shows the typical results of the flow cytometric analysis. The different staining patterns in this assay identify the different cell populations: region Q1 is damaged cells which are PI‐positive and annexin V‐negative; region Q2 is late apoptotic and dead cells which are PI‐positive and annexin V‐positive; region Q3 is early apoptotic cells which are PI‐negative and annexin V‐positive; region Q4 is vital cells which are PI‐negative and annexin V‐negative. As can be seen from Figure 6, the negative control (without being treated by the CD‐ALA‐TPP) exhibits high percentage (100%) of region Q4; while upon treatment with the CD‐ALA‐TPP and exposure to light irradiation, compared to the negative control, the percentage of vital cells decreases, but that for the late apoptotic/dead cells increases gradually in a dose‐dependent manner. And the number of late apoptotic cells increases from 0% for the control to 95.3% for the cell sample pretreated with 100 μg mL^−1^ CD‐ALA‐TPP. On the other hand, a comparison experiment was performed using a nanoparticle sample CD‐ALA (which has no TPP on its surface), and the result indicates that the number of late apoptotic cells only increases to 51.9% for the cell sample pretreated with 100 μg mL^−1^ CD‐ALA. These results clearly indicate that the ALA‐containing nanosystems can induce significant cell apoptosis upon light irradiation; and the targeted system can provide more efficient proapoptotic action toward cancer cells.

### 2.7 Cell Viability Assays for Two Cell Lines upon Different Treatments

**Figure 7:**
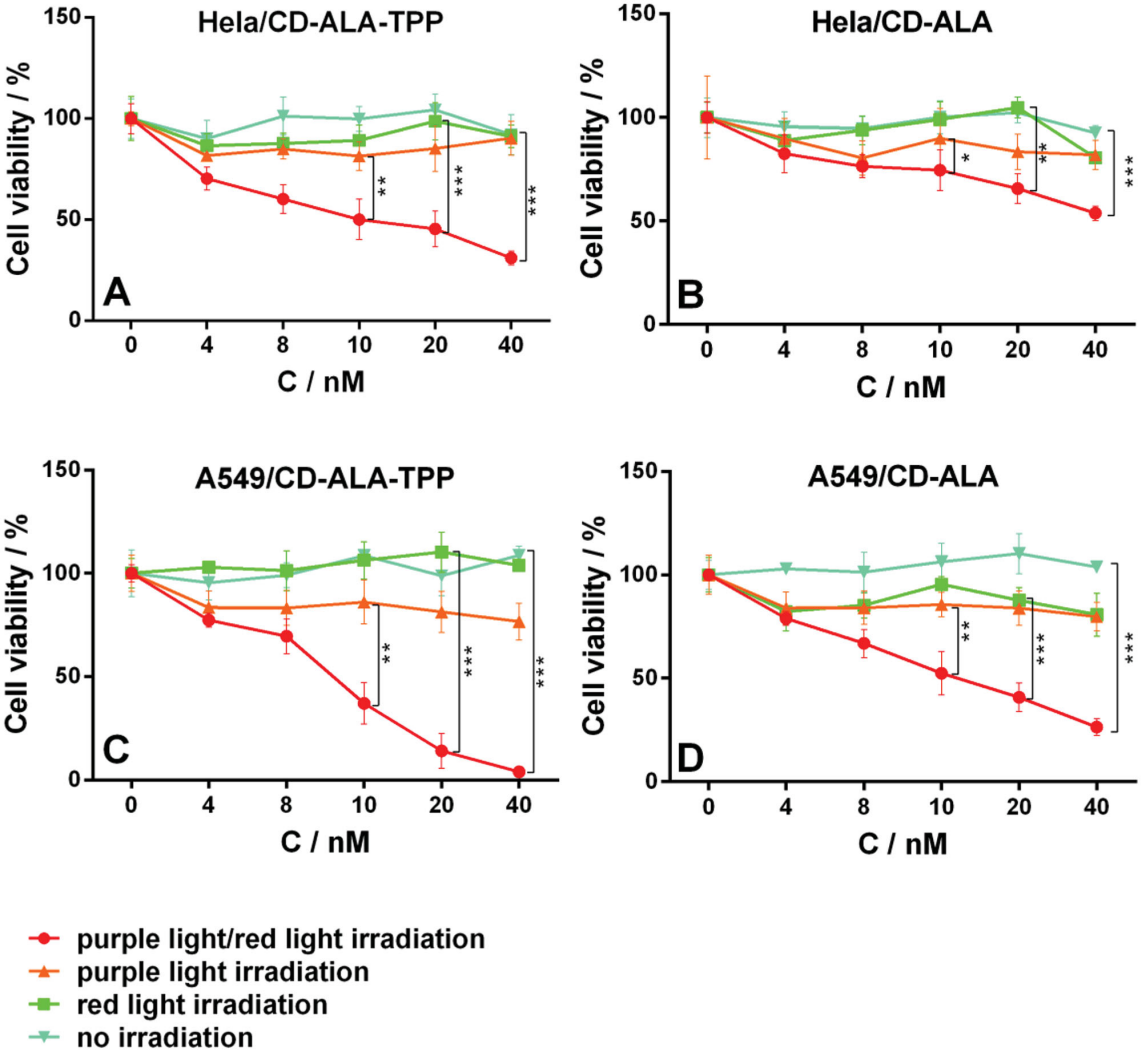
Cells viability of PDT systems for HeLa and A549 cell line. Cell viability was assessed by MTT assay upon 24 h of incubation after being treated with various concentrations of CD‐ALA‐TPP or CD‐ALA for 4 h (violet light and red light, violet light only, red light only, or no irradiation). The concentration *c* denotes the molar concentration of 5‐ALA moiety in nanosystems in the culture media. Each concentration was performed independently for three times, and for each independent experiment, the assays were performed in eight samples. The red light wavelength ranges from 645 to 655 nm and violet light from 400 to 450 nm, with the power density of 10 mW cm^−2^. Data represent mean ± SD from three independent experiments. *P < 0.05, **P < 0.01 and ***P < 0.001. A,B) Hela cells treated with CD‐ALA‐TPP and CD‐ALA respectivel; C,D) A549 cells treated with CD‐ALA‐TPP and CD‐ALA respectively.

To evaluate the efficacy of our nanosystems on the cell viability, we performed MTT assays for two cancer cell lines (HeLa and A549) upon exposure to CD‐ALA‐TPP or CD‐ALA. In addition, the effect of light irradiation on the cell viability was also investigated. As shown in **Figure** **7**, for the control samples (without being irradiated with any light), their viabilities barely change, suggesting the nanosystems exhibit no dark cytotoxicity. While for the samples irradiated with red light (from 645–655 nm) or violet light (from 400 to 450 nm) only, there is no significant reduction in cell viability; and compared to the red light, the violet light can lead to lower viability. These results reveal that the nanosystems are of low cytotoxicity before their cargo (5‐ALA) is released; and a single light (violet or red light) irradiation cannot induce remarkable cytotoxicity toward the cells. On the other hand, for the cells loaded with CD‐ALA‐TPP or CD‐ALA, significant decreases in cell viabilities are observed upon violet light and red light irradiation, as compared to those for the samples incubated in dark or after only one light irradiation. The CD‐based nanoparticles exhibit a dose‐dependent cytotoxicity; and the cells loaded with higher amount of nanoparticles have lower viability. In addition, the mitochondrion‐targeted system (CD‐ALA‐TPP) displays much higher cytotoxicity than the system with no TPP (CD‐ALA); and we suppose, with hydrophobic cations on its surface, CD‐ALA‐TPP can enter into cells more efficiently and preferably localize in mitochondria, thereby releasing 5‐ALA in close vicinity of mitochondria and promoting the latter's bioconversion into PPIX. For comparison, we performed MTT assays for HeLa cells treated with molecular 5‐ALA and then subject to light irradiations (Figure S16, Supporting Information), and we found that at the same concentration, molecular 5‐ALA exhibits much lower cytotoxicity than that of CD‐ALA‐TPP, indicating the current nanosystem is beneficial to the proapoptotic action. Moreover, the cell viability for the two cell lines upon exposure to red and/or violet light irradiation but not treated with the samples was also measured to further verify proapoptotic effect of CD‐ALA‐TPP, as shown in Figure S17 (Supporting Information). The results indicate that both cell lines exhibit high viability (above 80%) upon combined irradiation of red and violet light, proving that the nanosystem plays a vital role in the proapoptotic action toward cancer cells.

Compared with the previously reported CD‐based nanosystems,[[qv: 14c–e]] the carrier of the current system features moderate size (most CDs have the diameters ranging from 3 nm to 10 nm), green‐light emitting (majority of the CDs emit blue light), and relatively higher fluorescent quantum yield among green CDs; while the modification of the carrier (CDs) with TPP and ALA‐releasing element makes CD‐ALA‐TPP unique among the CD‐based nanosystems. The current nanosystem is the first nanosystem that targets mitochondria and releases bioprecursor of photosensitizer.

## 3 Conclusion

In summary, we have successfully developed a CD‐based PDT system featuring fluorescence imaging, mitochondria targeting, and two‐photon‐induced ALA releasing capability. The nanosystem can be readily internalized by the cells and localized in mitochondria. Upon one‐ or two‐photon irradiation, the nanosystem can release a PPIX bioprecursor (5‐ALA), which can be bio‐converted into mitochondrial PPIX. Upon red light irradiation, the PPIX generates singlet oxygen in mitochondria and induces the damage of the latter, thereby eventually leading to the efficient apoptosis of the cancer cells. Moreover, the targeted nanosystem can damage mitochondria more effectively and display enhanced apoptotic effect toward two cancer cell lines. We suppose this targeted and two‐photon activatable delivery system may potentially be exploited in PDT‐related applications.

## 4 Experimental Section

*Materials*: 1,4‐Dibromobutane, anhydrous K_2_CO_3_, trimethylamine, citric acid, and urea were supplied by Aladdin Reagents Co. Ltd. Methanol (MeOH) and N,N‐dimethylformamide were distilled before use. DCFH‐DA, p‐dimethylaminobenzaldehyde, and ethyl acetoacetate were purchased from Sigma‐Aldrich. The bovine serum albumin (BSA) and the culture medium RPMI1640 were obtained from Life Technologies. JC‐1 (5,5′,6,6′‐tetrachloro‐1,1′,3,3′‐tetraethyl benzimidazolylcarbocyanine iodide) and annexin V‐FITC/PI kit were purchased from the Beyotime Institute of Biotechnology. All other chemicals used were of analytical reagent grade. The water used in this study was the triple‐distilled water that was further treated by ion exchange columns and then by a Milli‐Q water purification system.

*Preparation of ALA‐Releasing Nanosystems*: The CD‐based nanosystems were prepared following the route presented in Scheme S1 (Supporting Information).

*Synthesis of Compound **1***: A mixture of 7‐hydroxy‐4‐methylcoumarin (300 mg, 1.7 mmol) and 1,4‐dibromobutane (2.045 mL, 17 mmol) was dissolved in 50 mL distilled acetonitrile. Potassium carbonate (2.35 g, 17 mmol) was then added to the solution and the mixture was refluxed for 3 d. After filtration, the solution was evaporated and the obtained crude product was purified by column chromatography on silica gel using EtOAc:Hexane = 1:1 to obtain the product in yield of 62.3% (330 mg). ^1^H NMR (600 MHz, CDCl_3_) *δ*: 7.40–7.36 (m, 1H), 6.48 (dd, *J* = 8.8, 2.4, 1H), 6.38 (d, *J* = 2.4, 1H), 5.93 (d, *J* = 0.8, 1H), 3.35 (t, *J* = 6.6, 4H), 2.34 (d, *J* = 1.0, 3H), 2.08–2.01 (m, 4H).

*Synthesis of Compound **2***: SeO_2_ (214.68 mg, 1.93 mmol) and compound **1** (300 mg, 1 mmol) were suspended in 20 mL p‐xylene, and the mixture was refluxed with vigorous stirring under argon atmosphere. After 24 h of reaction, the mixture was filtered and concentrated under reduced pressure. The obtained dark brown oil‐like product was dissolved in methanol (15 mL), and sodium borohydride (73.2 mg, 1.93 mmol) was added into the solution, which was then stirred for 6 h at room temperature. The resultant suspension was carefully neutralized with 1 m HCl, diluted with water, and then partially concentrated under reduced pressure to remove methanol. Afterward, the mixture was extracted with CH_2_Cl_2_ and the organic phase was collected and washed with water and brine, dried over Mg_2_SO_4_, and concentrated in vacuum. The obtained oil‐like product was purified by column chromatography (EtOAc:Hexane = 1:1) in yield 50.7% (157.7 mg). ^1^H NMR (600 MHz, DMSO) *δ*: 7.44 (d, *J* = 8.8, 1H), 6.59–6.48 (m, 1H), 4.67 (s, 2H), 3.31 (d, *J* = 6.2, 4H), 2.50 (dt, *J* = 3.4, 1.7, 2H), 2.05–1.88 (m, 4H).

*Synthesis of Coumarin‐ALA (Compound **3**)*: 4‐Nitrophenyl chloroformate (44.5 mg, 0.22 mmol) and 4‐dimethylaminopyridine (44.96 mg, 0.36 mmol) were dissolved in 20 mL distilled dichloromethane, which was then added with compound **2** (60 mg, 0.18 mmol) dissolved in CH_2_Cl_2_ (5 mL) drop by drop. The mixture was stirred for 1 h at room temperature, and the resultant ester intermediate was immediately allowed to react with 5‐ALA (48.25 mg, 0.36 mmol) in dry CH_2_Cl_2_ (5 mL) containing 1 mL dry Et3N. After 2 h of reaction, the solvent was removed under reduced pressure. The product was purified by column chromatography (Hexane:EtOAc = 1:1) in yield 68.9% (60 mg). ^1^H NMR (600 MHz, CDCl_3_) *δ* = 8.42 (s, 1H), 8.27–8.14 (m, 1H), 6.65 (d, *J* = 7.2, 2H), 5.50 (s, 1H), 3.58 (q, *J* = 7.3, 2H), 3.48 (q, *J* = 7.2, 2H), 3.14 (s, 4H), 3.07 (dt, *J* = 10.8, 5.3, 2H), 2.77–2.69 (m, 2H), 1.44–1.39 (m, 2H), 1.37–1.30 (m, 2H).

*Preparation of CDs*: The preparation of C‐dots was performed following a literature‐reported procedure.^14^ Briefly, citric acid (3 g) and urea (6 g) were added to distilled water (10 mL) to form a transparent solution. The solution was then heated in a 650 W microwave oven for 4–5 min, during which the solution changed from colorless liquid to brown and finally dark‐brown clustered solid, indicating the formation of C‐dots. The resultant solid was transferred to a vacuum oven and heated at 60 °C for 1 h. The dispersion of the crude product was dropped into excessive ethanol aqueous solution (ethanol volume concentration: 65%) and stirred for 10 min, followed by centrifugation (3000 r min^−1^, 20 min) to remove large or agglomerated particles.

*Preparation of CD‐ALA‐TPP*: Compound **3** (20 mg), potassium carbonate (200 mg), and C‐dots (100 mg) were dissolved in 15 mL distilled N,N‐Dimethylformamide, and the mixture was stirred for 24 h at 95 °C. Thereafter, to the resultant solution 3‐bromopropyl‐ triphenylphosphonium bromide (20 mg) was added. After 24 h of reaction, the mixture was filtered to remove excessive K_2_CO_3_, and the dispersion of crude product was dropped into ethyl acetate:ethanol = 1:6 aqueous solution and stirred for 10 min, followed by centrifugation (6000 r min^−1^, 10 min) to remove large or agglomerated particles.

*Preparation of CD‐ALA*: Compound **3** (20 mg), potassium carbonate (200 mg), and C‐dots (100 mg) were dissolved in 15 mL distilled N,N‐Dimethylformamide, and the mixture was stirred for 24 h at 95 °C. After 24 h, the mixture was filtered, and the dispersion of crude product was dropped into ethyl acetate:ethanol = 1:6 aqueous solution and stirred for 10 min, followed by centrifugation (6000 r min^−1^, 10 min) to remove large or agglomerated particles.

*Characterization*: ^1^H NMR spectra were recorded on a Bruker Avance 600 MHz NMR spectrometer. Confocal laser scanning microscopy (CLSM) images were collected using a Leica TCS‐SP5 confocal microscope. Single photon fluorescence spectra were recorded on a Hitachi F‐4600 fluorescence spectrophotometer. Two‐photon fluorescence was measured on an experimental setup described previously;^15^ and a Coherent Legend Elite Ti:sapphire regenerative amplifier system as the femtosecond laser source, which emitted laser pulses with a repetition rate of 1 kHz, a pulse width of 130 fs, and a central wavelength of 800 nm. UV–vis spectra were recorded on a Hitachi U‐3010 UV–vis spectrophotometer. High‐resolution transmission electron microscopy (HR‐TEM) was performed on a JEM‐2100HR electron microscope. Dynamic light scattering measurement was carried out on a Malvern Nanosizer. Flow cytometry were recorded on a BD CS6 flow cytometer.

*In Vitro Release of ALA from Nanosystem*: The in vitro release of ALA was assayed by absorption spectrometry upon one‐photon irradiation (LED lamp, with the wavelength range of 400–450 nm at the power of 10 mW cm^−2^) or two‐photon irradiation (Coherent Legend Elite Ti:sapphire regenerative amplifier system as the femtosecond laser source, 800 nm). The mechanism for the assay is given in Scheme S2 (Supporting Information): the released 5‐ALA can react with ethyl acetoacetate to produce a pyrrole derivative, which can be extracted by ethyl acetate and then react with p‐dimethylaminobenzaldehyde to generate a red compound with a maximum absorption wavelength at 554 nm.

The chromogenic reagent for the assay was prepared in advance by mixing 30 mL of acetic acid, 1 g of p‐dimethylaminobenzaldehyde, 5 mL of perchloric acid, and 5 mL of water, followed by diluting the solution into 50 mL acetic acid. The samples for the assay were prepared by dispersing 20 mg of CD‐ALA‐TPP into 2 mL distilled water, followed by the addition of 0.4 mL ethyl acetoacetate into the dispersion. The samples were then subject to light irradiation for different time periods. After light irradiation, each sample was extracted with 1 mL of ethyl acetate, and the extract was added into 1 mL chromogenic reagent. The amount of released ALA was determined by recording the absorbance at 554 nm. The 5‐ALA release experiment was performed in triplicate.

*Cell Culture*: HeLa cells were cultured in complete medium containing Dulbeco's modified Eagle's medium (DMEM), supplemented with 10% fetal bovine serum (FBS) and 1% penicillin and streptomycin at 37 °C, 5% CO_2_; while A549 cells were cultured in complete medium containing RPMI1640, supplemented with 10% FBS and 1% penicillin and streptomycin at 37 °C, 5% CO_2_.

*Cell Imaging*: The cellular uptake and co‐localization experiments were observed on a CLSM. HeLa cells were grown on glass slides in 6‐well plates. After 24 h of growth, cells were treated with CD‐ALA‐TPP with varied concentrations ranging from 20 μg mL^−1^ to 100 μg mL^−1^. Before imaging, cells (on glass slides) were washed with DMEM, and incubated in DMEM medium containing JC‐1 dye for 30 min. Finally, the cells which grew on glass slides were placed on a Leica TCS SP5 CLSM for imaging.

*Annexin V‐FITC/Propidium Iodide Assay*: HeLa cells were seeded in 6‐well plates (1.0 × 10^6^ cells per dish) at 37 °C and then incubated for 12 h. Afterward the cells were incubated in the media containing varied amounts of CD‐ALA‐TPP (20 μg mL^−1^, 40 μg mL^−1^, 50 μg mL^−1^, or 100 μg mL^−1^) for 4 h, and the cells were then subject to 30 min of one‐ (400–450 nm, 10 mW cm^−2^) or two‐photon (800 nm) irradiation, allowing for release of ALA from the nanosystem. After 2 h incubation, the cells were exposed to red light irradiation (645–655 nm, 10 mW cm^−2^) for 15 min, allowing for generation of singlet oxygen. After being washed by phosphate buffered saline (PBS), trypsinized, and centrifuged, the cells were treated with Annexin V‐FITC/Propidium Iodide (PI) kit for flow cytometry assay.

*Tracking the Variation in Mitochondrial Membrane Potential*: Flow cytometry was utilized to track the variation in mitochondrial membrane potential with JC‐1 dye as the fluorescent indicator. After HeLa cells were treated with the nanosystems, one‐photon process and then the red light irradiation as described above, the cells were washed three times with PBS, and then detached with trypsin–ethylenediaminetetraacetic acid (EDTA) solution. The resultant cell suspensions were incubated for 30 min with 1 μg mL^−1^ of JC‐1 in DMEM complete medium at 37 °C in the dark. After being washed with PBS and centrifugation for three times, the cells were resuspended in 1 mL of PBS with 2% BSA for flow cytometry assay.

*Dark Cytotoxicity Assay*: The viability of HeLa and A549 cells treated with CD‐ALA‐TPP or CD‐ALA were assessed by MTT assay. The cells were transferred into 96‐well plates at the cell population of >>5000 cells per well. After 24 h of incubation, cells were washed with PBS buffer, and the PBS was replaced with fresh complete medium containing CD‐ALA‐TPP or CD‐ALA (nanoparticle content: 0–200 μg mL^−1^, corresponding to 0–40 × 10^−9^ m of 5‐ALA moiety), then the samples were subject to incubation for 24 h. Thereafter, the cells were washed with PBS buffer and incubated for another 4 h with DMEM (HeLa) or RPMI 1640 (A549) medium containing 0.5 mg mL^−1^ MTT. After removing the culture medium, 150 μL of DMSO was added to dissolve the precipitates and the absorbance was read on a Thermo MK3 ELISA reader at 570 nm. As for the assays, each concentration was performed independently for three times, and for each independent experiment, the assays were performed in eight samples. And the statistical mean and standard deviation were used to estimate the cell viability.

*Cell Viability Assay upon Light Irradiation*: The HeLa and A549 cells were first treated with the nanosystems for 4 h, irradiated by violet light (400–450 nm) irradiation (30 min), after 2 h of incubation, the cells were exposed to red light (645–655 nm) irradiation for 15 min as described above, and the cells were incubated for another 17 h 15 min for MTT assay.

## Supporting information

As a service to our authors and readers, this journal provides supporting information supplied by the authors. Such materials are peer reviewed and may be re‐organized for online delivery, but are not copy‐edited or typeset. Technical support issues arising from supporting information (other than missing files) should be addressed to the authors.

Supplementary
