# Supplementary material for: A Nanosystem Capable of Releasing a Photosensitizer Bioprecursor under Two‐Photon Irradiation for Photodynamic Therapy
Source: Adv Sci (Weinh). 2015 Nov 25;3(2):1500254. doi: 10.1002/advs.201500254 (PMC5063179; doi:10.1002/advs.201500254)
Supplement: Supplementary file 1 — Supplementary [file ADVS-3-0k-s001.pdf]

Copyright WILEY-VCH Verlag GmbH & Co. KGaA, 69469 Weinheim, Germany, 2015.

ADVANCED SCIENCE
Open Access

Supporting Information

for *Adv. Sci.*, DOI: 10.1002/advs. 201500254

A Nanosystem Capable of Releasing a Photosensitizer Bioprecursor under Two-Photon Irradiation for Photodynamic Therapy

Hao Wu, Fang Zeng, Hang Zhang, Jiangsheng Xu, Jianrong Qiu, and Shuizhu Wu**

Copyright WILEY-VCH Verlag GmbH & Co. KGaA, 69469 Weinheim, Germany, 2015.

Supporting Information

A Nanosystem Capable of Releasing a Photosensitizer Bioprecursor under Two-photon Irradiation for Photodynamic Therapy

Hao Wu, Fang Zeng, Hang Zhang, Jiangsheng Xu, Jianrong Qiu, Shuizhu Wu**

Photorelease of ALA and quantification of released ALA.

(A)

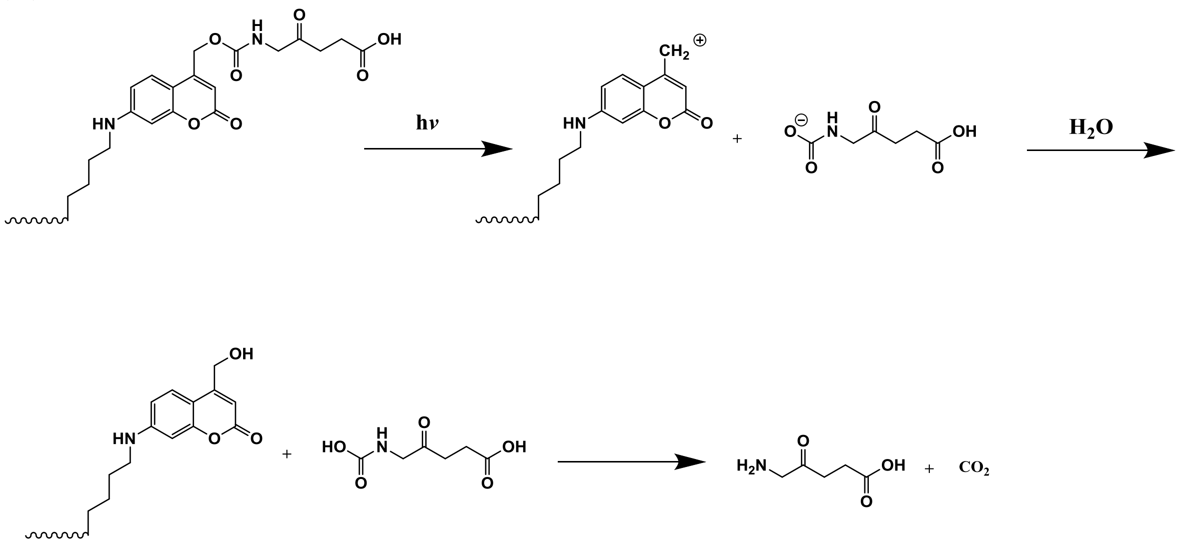

(B)

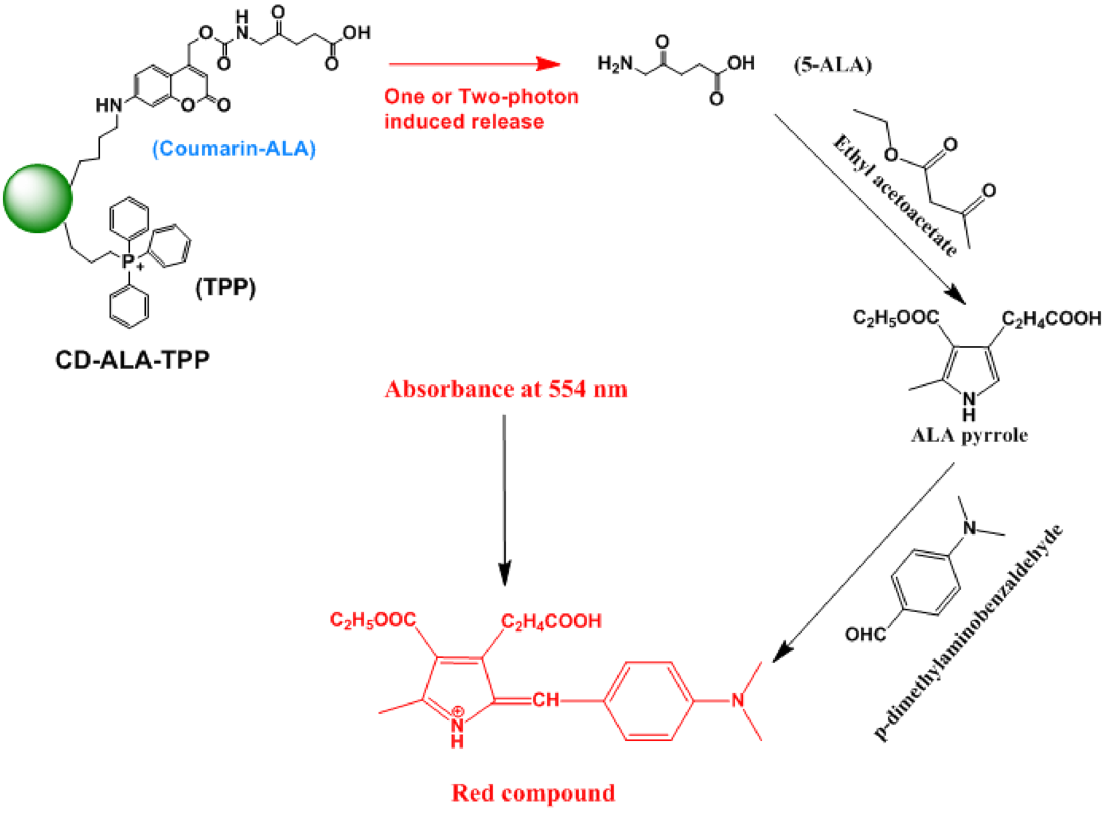

Scheme S1. Light-induced release (A) and quantification (B) of 5-ALA. 5-aminolevulinate was determined colorimetrically at 554 nm with modified Ehrlich's reagent following its quantitative conversion to ALA pyrrole with ethyl acetoacetate. The reaction routes are illustrated according to literature reports.

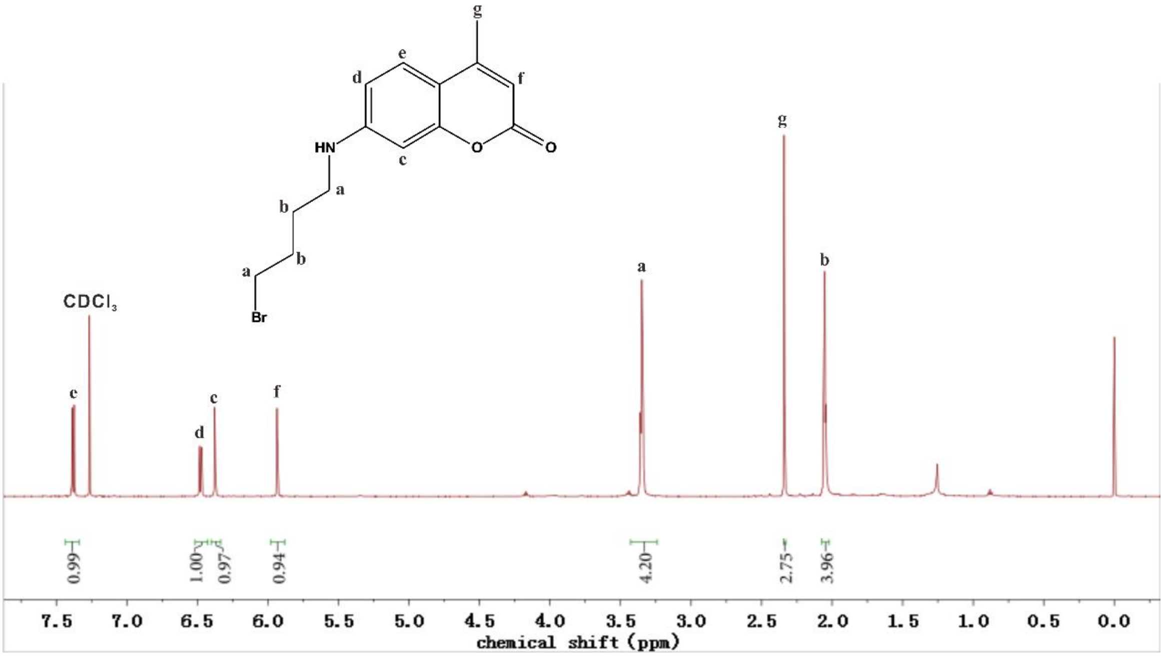

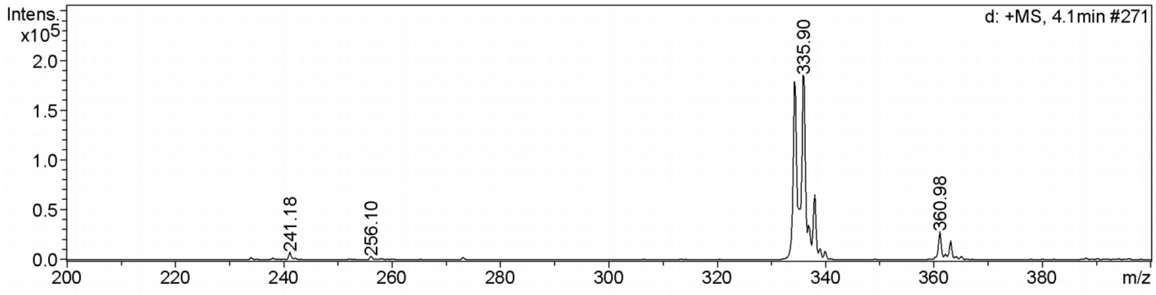

Figure S1. ^{1}H NMR spectrum (in $CDCl_3$) and mass spectrum for compound **1**. MS (ESI): m/z 335.90 $[M+Na]^+$.

The peak at 1.25 ppm and 0.88 ppm in ^{1}H NMR spectrum correspond to the methylene and methyl protons of residual high-boiling-point aliphatic hydrocarbons which come from the eluent petroleum ether (same as below).

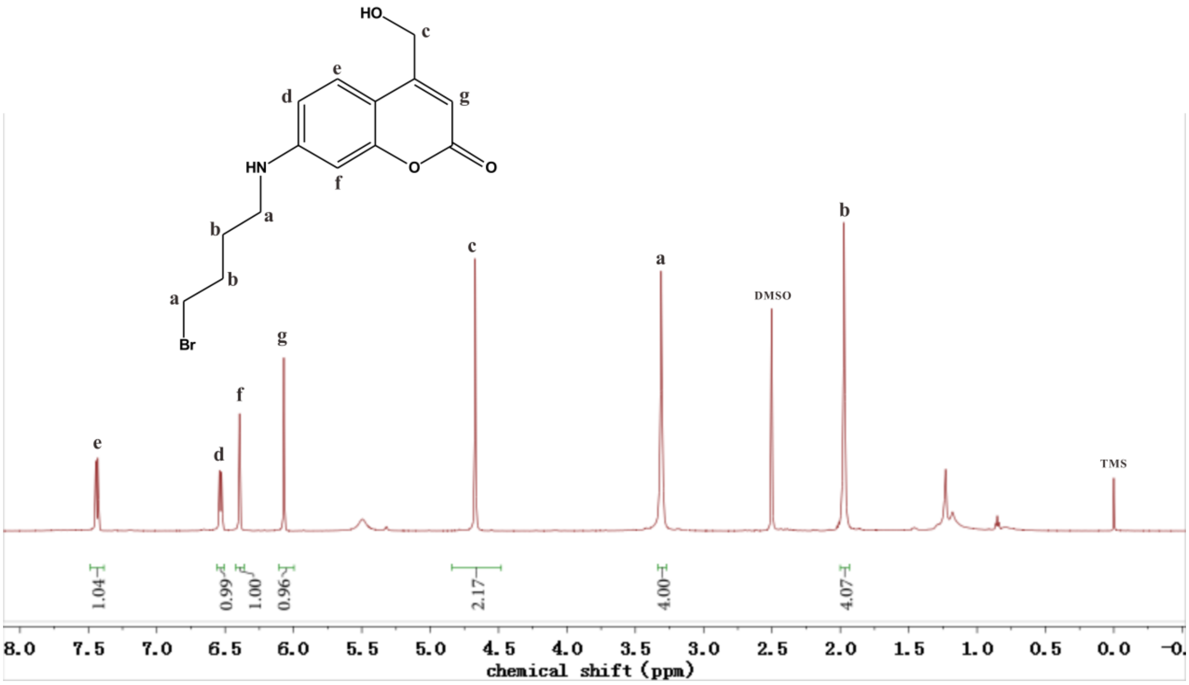

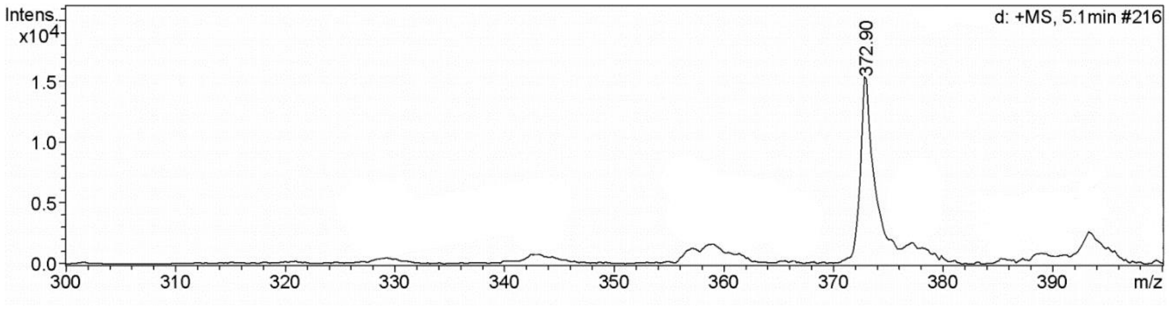

Figure S2. ^{1}H NMR spectrum (in deuterated DMSO) and mass spectrum for compound **2**. MS (ESI): m/z 372.90 $[M+K]^{+}$.

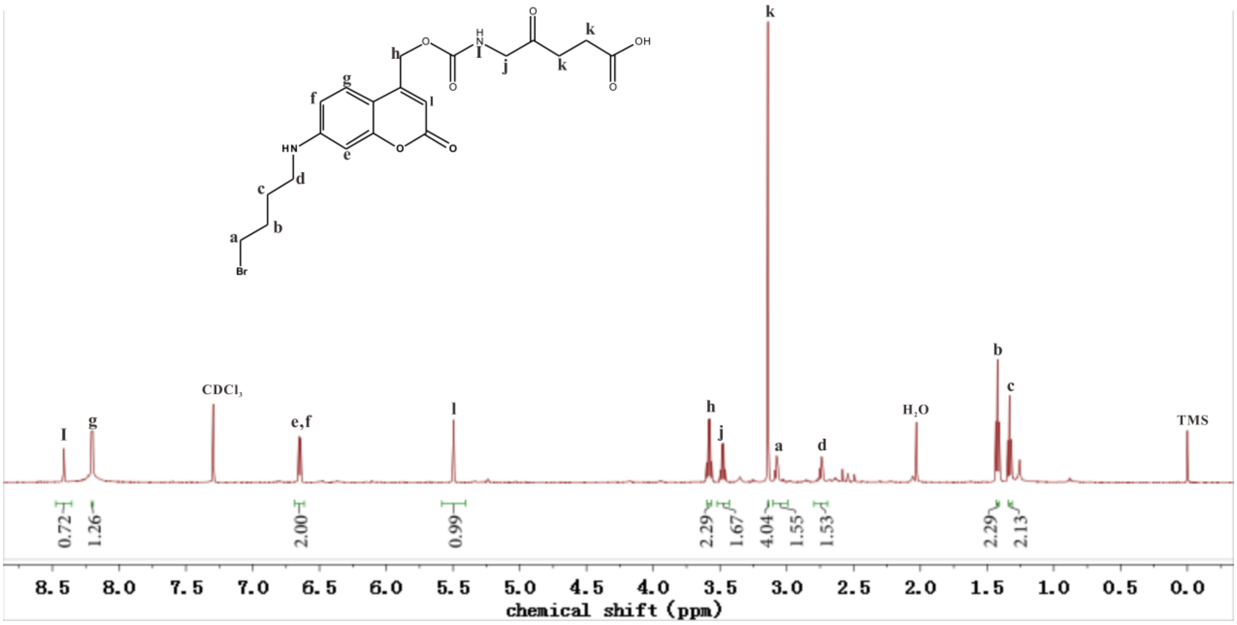

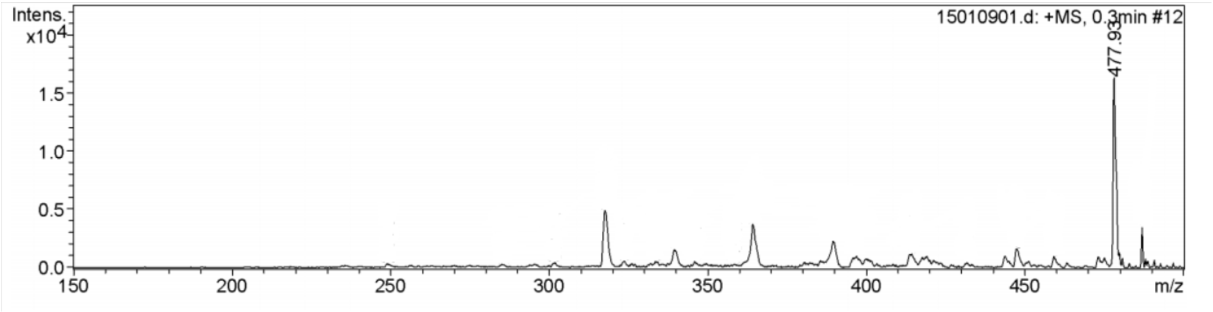

Figure S3. ^{1}H NMR spectrum (in $CDCl_3$) and ESI mass spectrum for compound **3**. MS (ESI): m/z 447.93 $[M+H]^+$.

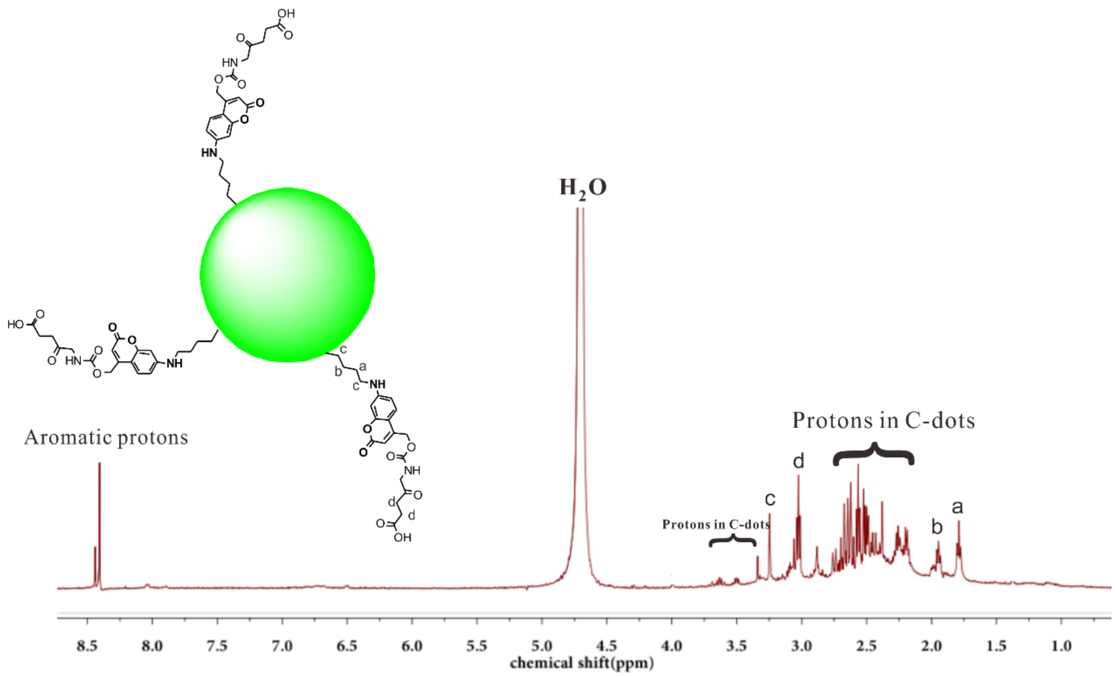

Figure S4. ^{1}H NMR spectrum (in D_2O) for CD-ALA.

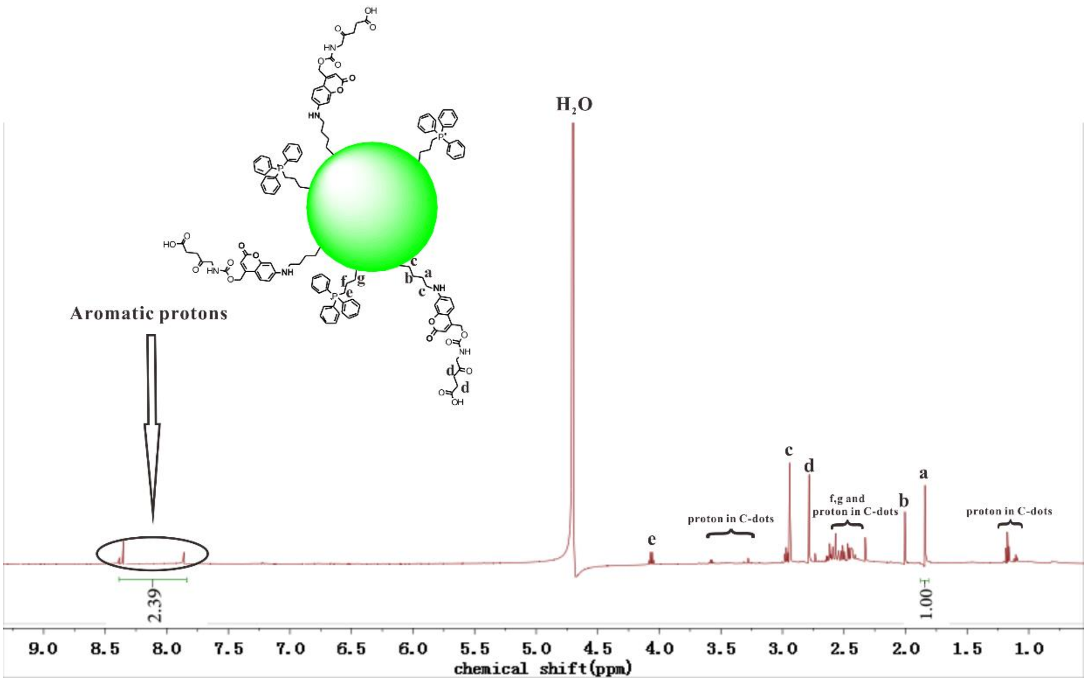

Figure S5. ^{1}H NMR spectrum (in D_2O) for CD-ALA-TPP.

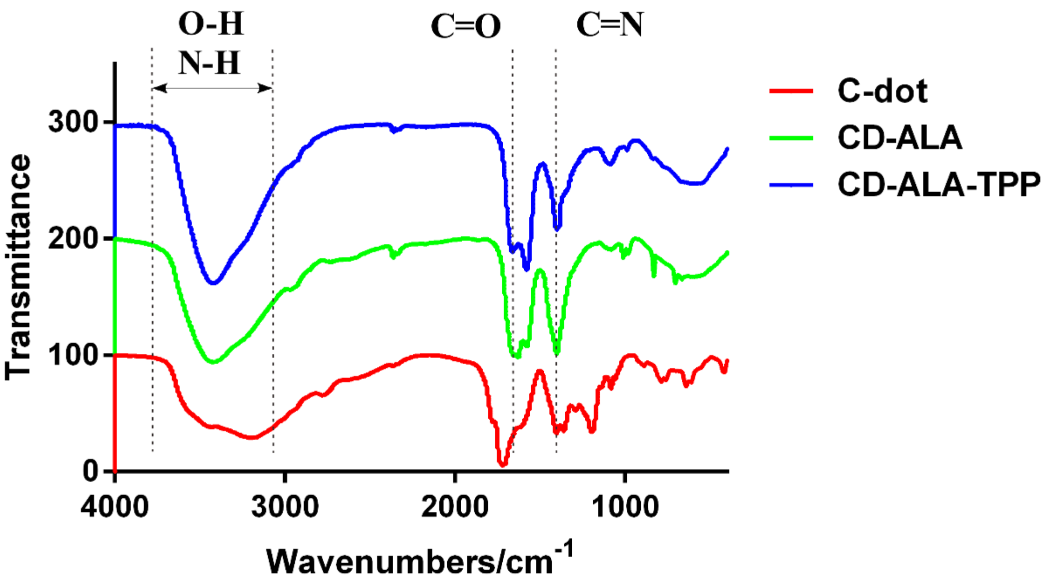

Figure S6. FT-IR spectra of C-dot (red line), CD-ALA (green line) and CD-ALA-TPP (blue line) in their dry (powder/KBr) state.

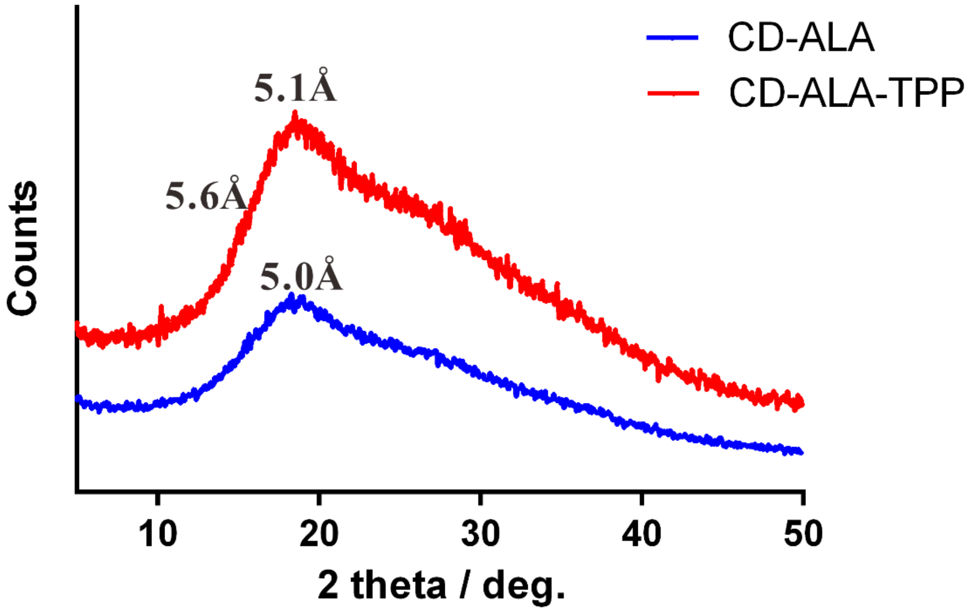

Figure S7. XRD patterns of CD-ALA (blue line) and CD-ALA-TPP (red line) in their dry (powder) state.

Determination of two-photon absorption cross-section.

In this study, two-photon induced fluorescence (TPIF) intensity was obtained and used to determine the two-photon absorption cross-section of CD-ALA-TPP.[1] A 3.82 × 10 $^{-5}$ M (18.3 mg/L) rhodamine B solution (in methanol) was used as the reference, with its two-photon absorption cross-section of 150 GM in methanol.[2] A CD-ALA-TPP/methanol solution with its concentration of 18.3 mg/L was used as the sample for cross-section determination. Since the input beam parameters are the same for all experiments, we can calculate the two-photon absorption cross-section, δ_2, of CD-ALA-TPP using the known two-photon absorption cross-section, δ_1, of rhodamine B molecule as the reference:

$$\delta_2 = \delta_1 \left(\frac{F_2}{F_1}\right)\left(\frac{\varphi_1}{\varphi_2}\right)\left(\frac{c_1}{c_2}\right)$$

where F_1 and F_2 are the integrated fluorescence intensities measured at the same power of the excitation beam; φ_1 and φ_2 are the fluorescence quantum yield for the standard reference (Rhodamine B) and for the coumarin derivative (for subsequent release of ALA) in the nanosystem, respectively; c_1 and c_2 are the concentrations (index 1 corresponds to Rhodamine B, index 2 is used for CD-ALA-TPP).

Fluorescence quantum yields φ_2 was measured as 0.253 according to the literature.[3] Quinine sulfate (φ= 0.54) was dissolved in 0.1 M H_2SO_4 as the standard material for the measurement.

Eventually, the two-photon absorption cross-section of CD-ALA-TPP was determined as 303.4 GM at 800 nm.

References:
[1] J. Hermann, J. Ducuing, *Opt. Commun.* **1972**, *6, 101.*
[2] G. A. Crosby, J. N. Demas, *J. Phys. Chem.* **1971**, *75, 991.*
[3] a) H. J. Yvon, HORIBA, Jobin Yvon Ltd., Stanmore, Middlesex, UK **2012**; b) F. Du, F. Zeng, Y. Ming, S. Wu, *Microchim. Acta.* **2013**, *180, 453.*

Determination of fluorescence quantum yields for the C-dots.

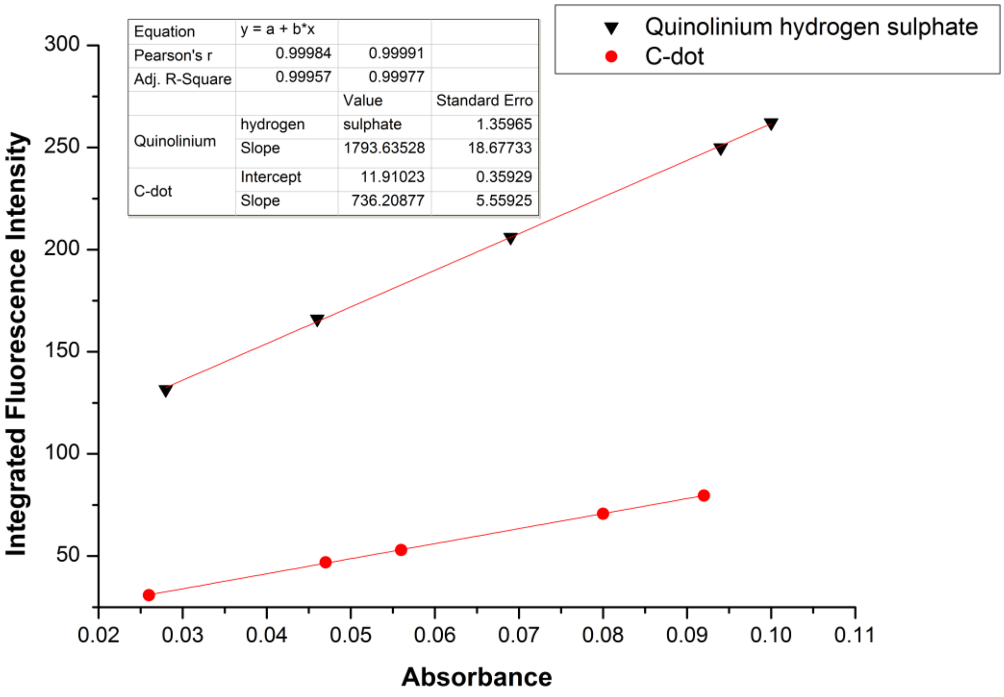

Figure S8. Fluorescence intensity and absorbance of C-dots and quinolinium sulphate.

The quantum yield (φ) of the C-dots was calculated by comparing their integrated fluorescence intensities (excitation at 360 nm) and absorbance values at 360 nm with those of quinine hydrogen sulfate. Quinine sulfate (φ_{ST}= 0.54) was dissolved in 0.1 M H_2SO_4 (refractive index: 1.33) and the C-dots were dissolved in water (refractive index: 1.33). The integrated fluorescence intensity is the area under the fluorescence curve in the wavelength range from 380 to 600 nm. Quantum yield can be calculated according to the following equation:

$$\varphi_X = \varphi_{ST}\left(\frac{Grad_X}{Grad_{ST}}\right)\left(\frac{\eta_X^2}{\eta_{ST}^2}\right)$$

Where the subscripts ST and X denote standard (quinine sulfhate solution) and test respectively, φ is the fluorescence quantum yield, Grad the gradient from the plot of integrated fluorescence intensity vs absorbance, and η the refractive index of the solvent. In

order to minimize the re-absorption effects, absorbance values in the 10 mm fluorescence cuvettes should be maintained under 0.1 at the excitation wavelength. Excitation and emission slit widths were set at 5.0 nm when recording their fluorescence spectra. The fluorescence quantum yields of C-dots was determined as 0.22.

Determination of photolysis quantum yield of CD-ALA-TPP

The experiments were carried out using a previously reported method [1]. 10 mg of CD-ALA-TPP was dissolved in 10 mL water in quartz cuvette, and was then irradiated under violet light (from 400 to 450 nm). Based on the release profile data for 5-ALA as shown in **Figure 2**, we plotted normalized released amount versus irradiation time. Further, the quantum yield for the photolysis of coumarin in CD-ALA-TPP was calculated using the following equation:

$$\emptyset_p = \frac{(k_p)_s V_s}{I_0(F_S)}$$

where the subscript s denotes CD-ALA-TPP. Φ_p is the photolysis quantum yield, V_s is the reaction volume (10 mL). k_p is the photolysis rate constant, which is mesaured as 1.02×10^{-9} mol L^{-1} s^{-1} acording to **Figure 2,** and I_0 is the incident photon flux which is 3.12×10^{-9} einstein $cm^{-2}s^{-1}$, and F is the fraction of light absorbed. Potassium ferrioxalate was used as an actinometer. Photolysis quantum yield of CD-ALA-TPP is calculated as 0.327%.

[1] a) M. Gangopadhyay, S. K. Mukhopadhyay, S. Karthik, S. Barman, N. D. P. Singh, *Med. Chem. Commun*, **2015**, *6, 769*; b) Q. Zhang, C. Li, T. Li, *Int. J. Photoenergy*, **2012**, *149, 251*.

Determination of ALA and TPP contents on carbon dots:

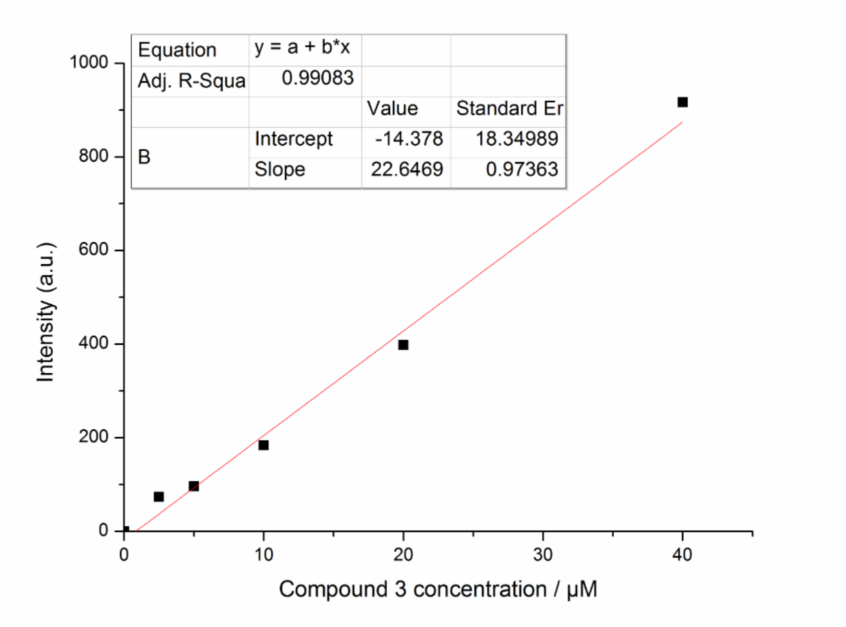

Figure S9. Relationship between concentration for compound **3** (coumarin-ALA) and the fluorescence intensity at 460 nm.

(A) Calculation of coumarin-ALA content:

First, 10 mg dried CD-ALA-TPP was dissolved in 2 mL of DMSO to form a dispersion with its concentration of 5 mg/mL, then the fluorescence intensity at 460 nm was measured as 1.88.

The calibration curve of coumarin-ALA in DMSO (as shown in Figure S10) was determined as Y = -18.66 + 22.333X (Y represents the fluorescence intensity, X the coumarin-ALA concentration). With Y=1.88, X can be calculated as 0.92 μmol/L (or 444.65 μg/mL).

Thus, the content of coumarin-ALA was calculated as 88.93 mg/g (Cdots), and accordingly the ALA content was determined as 24.13 mg/g (or 2.4 wt%).

(B) Calculation of TPP content:

The TPP content can be calculated based on the content of coumarin-ALA and the ^{1}H

NMR spectrum of the nanosystem (Figure S5):

$$\mathrm{TPP(wt\%)} = \frac{\frac{S_1}{15}}{\frac{S_3}{2}} \times (-\mathrm{compound\ 3}) \times 319$$

$$\mathrm{TPP(wt\%)} = \frac{\frac{S_1}{15}}{\frac{1}{2}} \times \frac{88.93}{403.32} \times 319$$

$$\frac{-\mathrm{compound\ 3}}{\frac{S_2}{3}} = \frac{-\mathrm{compound\ 3}}{\frac{S_3}{2}}$$

$$S_2 = 1.5$$

$$S_1 + S_2 = 2.39$$

$$S_1 = 0.89$$

$$\mathrm{TPP(wt\%)} = 8.34\ \mathrm{mg/g}$$

where S_1 is the integrated peak areas of the aromatic protons of TPP; S_2 the integrated peak area of aromatic protons of compound **3**; S_1+S_2 represents the integrated peak area of aromatic protons are 2.39; S_3 is the integrated peak area of ethyl protons of coumarin-ALA; while "-compound 3" represents the calculated content of compound **3** (coumarin-ALA); and 319 is the molecular weight of TPP; 403.32 is the molecular weight of coumarin-ALA.

Determination average numbers of ALA and TPP on a single carbon dot:

1. Estimated volume of a particle:

C-dots (volume)= $\frac{4}{3} \times \pi R^3$== $\frac{4}{3} \times \pi \times 2.5^3$ nm^3=65.42 nm^3

2. Estimated weight of a particle:

C-dots (weight)= C-dots (volume) × C-dots (density)=65.42 × 10^{-21} cm^3 × 1.06 g/cm^3=6.93×10^{-20} g

3. Average number of nanoparticle per gram:

   C-dots (number/g)=$\frac{1}{\text{C−dots (weight)}}$=$1.44\times10^{19}$/g

4. Estimated ALA number per gram nanoparticle:

   ALA(numbers/g)= $\frac{\text{ALA content}}{\text{ALA (molecular weight)}}\times$ Avogadro constant= $\frac{24.13\text{x}10^{-3}\text{g}}{131.13\text{g/mol}}\times$

   6.02×10^{23}=1.11×10^{20}/g

5. Average number of ALA on a C-dots = $\frac{\text{ALA(numbers/g)}}{\text{C−dots (numbers/g)}}$ = $\frac{1.11\text{x}10^{20}}{1.44\text{x}10^{19}}$= 7.7

6. Number of TPP per gram nanoparticle:

   TPP(numbers/g)= $\frac{\text{TPP content}}{\text{TPP (molecular weight)}}\times$ Avogadro constant = $\frac{8.34\text{x}10^{-3}\text{g}}{319\text{g/mol}}\times$

   $6.02\text{x}10^{23}$=1.57×10^{19}/g

7. Average numbers of TPP on a C-dot = $\frac{\text{TPP(numbers/g)}}{\text{C−dots (numbers/g)}}$ = $\frac{1.57\text{x}10^{19}}{1.44\text{x}10^{19}}$ =1.09

Note: Since there is no report on the C-dot's density, the density of a nanosized carbon material graphene (1.06 g/cm^3) [1] is used instead. The average radius for the nanosystem R is 2.5 nm accroding to the DLS result. The average numbers of 5-ALA on the C-dots was thereofore calculated as 7.7, and that of TPP on the C-dots was calculated as 1.09.

The [TPP]:[ALA] ratio is therefore calculated as: 1.09: 7.7 = 1:7.

Referenece:

[1] M. A. Rafiee, J. Rafiee, Z. Wang, H. Song, Z. Z. Yu, N. Koratkar, *Acs Nano.* **2009**, *3, 3884.*

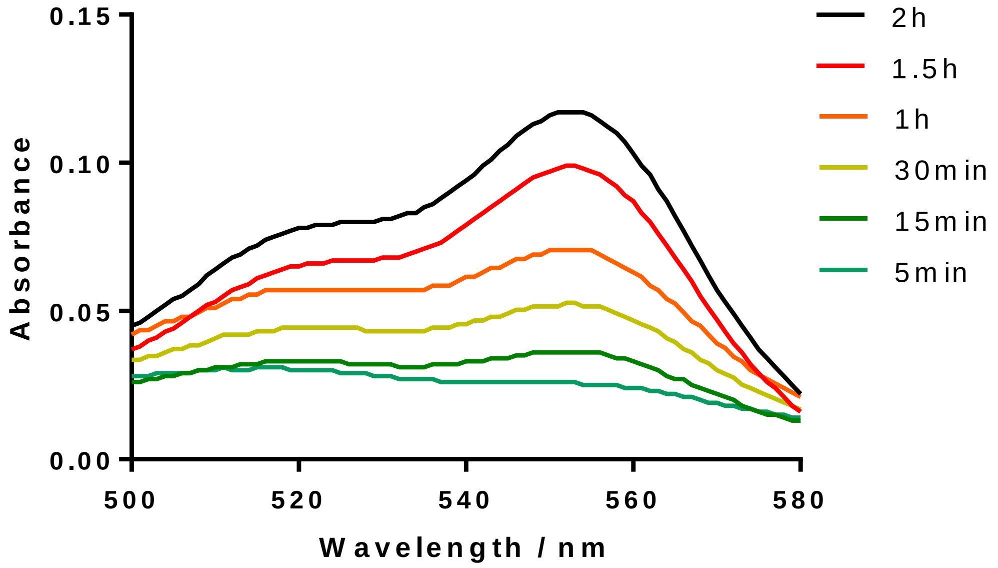

Figure S10. Absorption spectra for the released 5-ALA treated with chromogenic agent after one-photon irradiation.

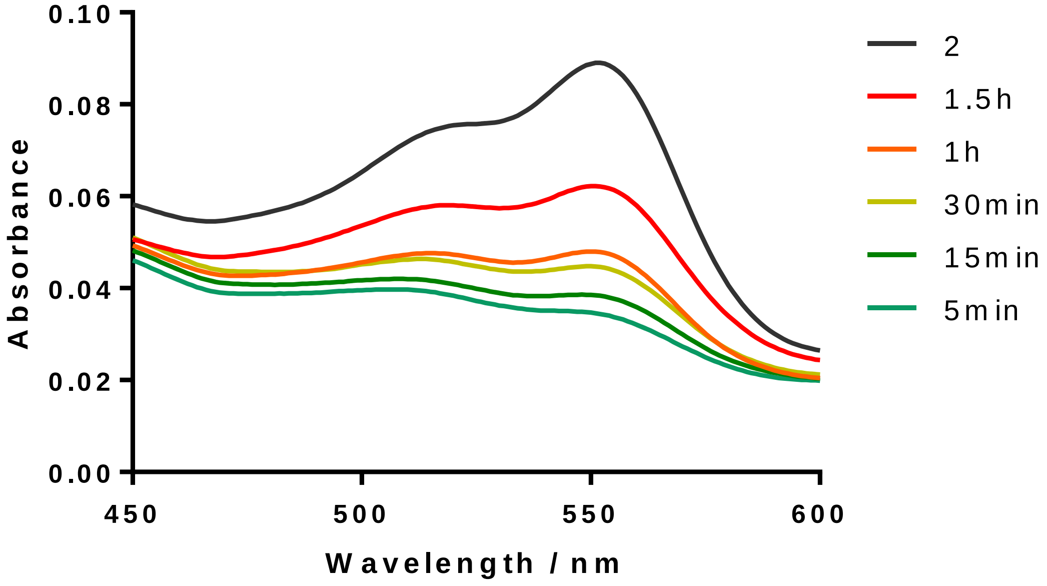

Figure S11. Absorption spectra for the released 5-ALA treated with chromogenic agent after two-photon irradiation.

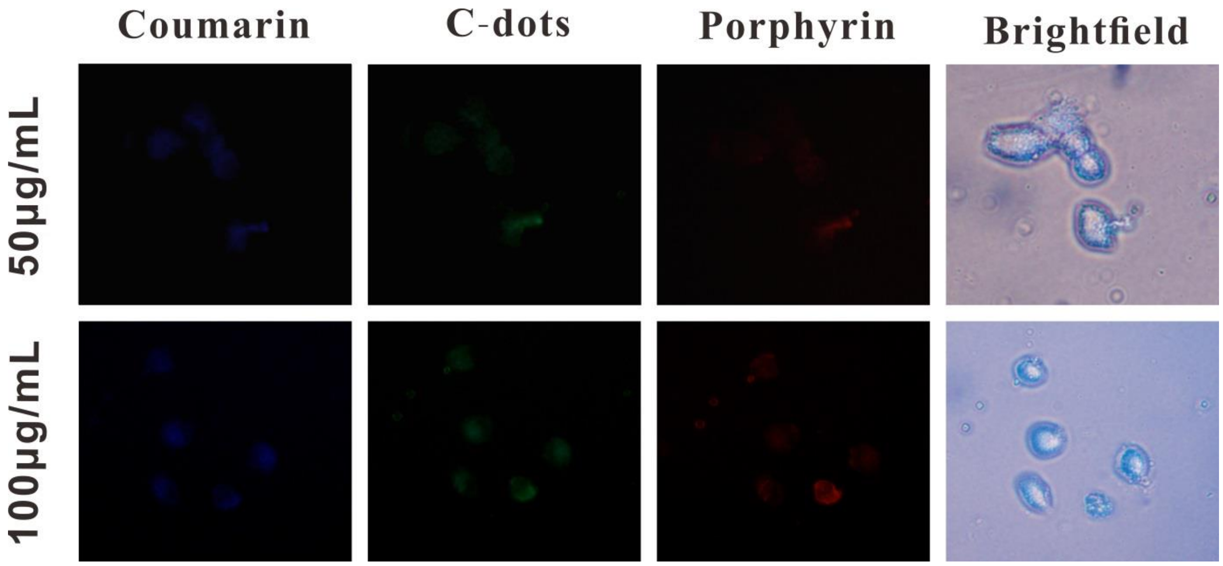

Figure S12. Fluorescence microscopic images for Hela cells stained with CD-ALA-TPP at varied concentrations (subject to 30 min of 400 - 450 nm light irradiation so as to trigger the release of ALA).

Determination of quantity of PPIX produced in HeLa cells as the result of nanosystem treatment. (By using a ELISA kit, Human FEP)

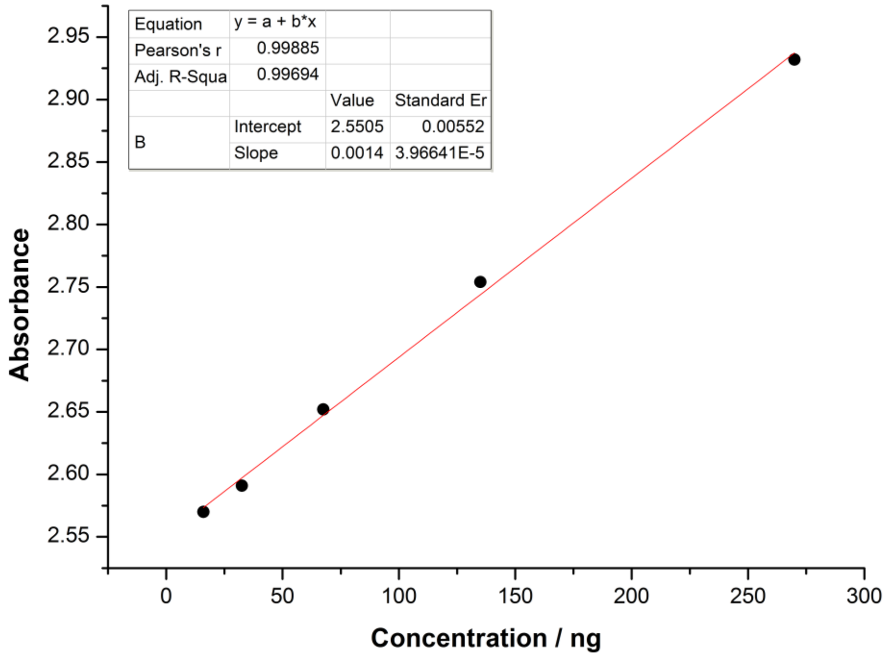

Figure S13. A standard curve prepared with standard PPIX samples using a ELISA kit Human FEP.

For this assay, Hela cells were first incubated in the media containing 200 μg/mL CD-ALA-TPP, and the treated Hela cells (about 2×10^5 cells) were homogenized in 4 mL of Tris-HCL (50 mM)/ ethanol (9:1), sonicated for 20 min, and then centrifuged for 30 min. The supernatant was diluted 400 times and used as the sample for the assay. Detection of PPIX concentration using the ELISA kit was conducted according to the instructions of the ELISA kit, which involves 11 steps. The OD value for the supernatant was measured as 2.65, and the production of PPIX in cells was calculated as 664 nM/ 10^5 cells.

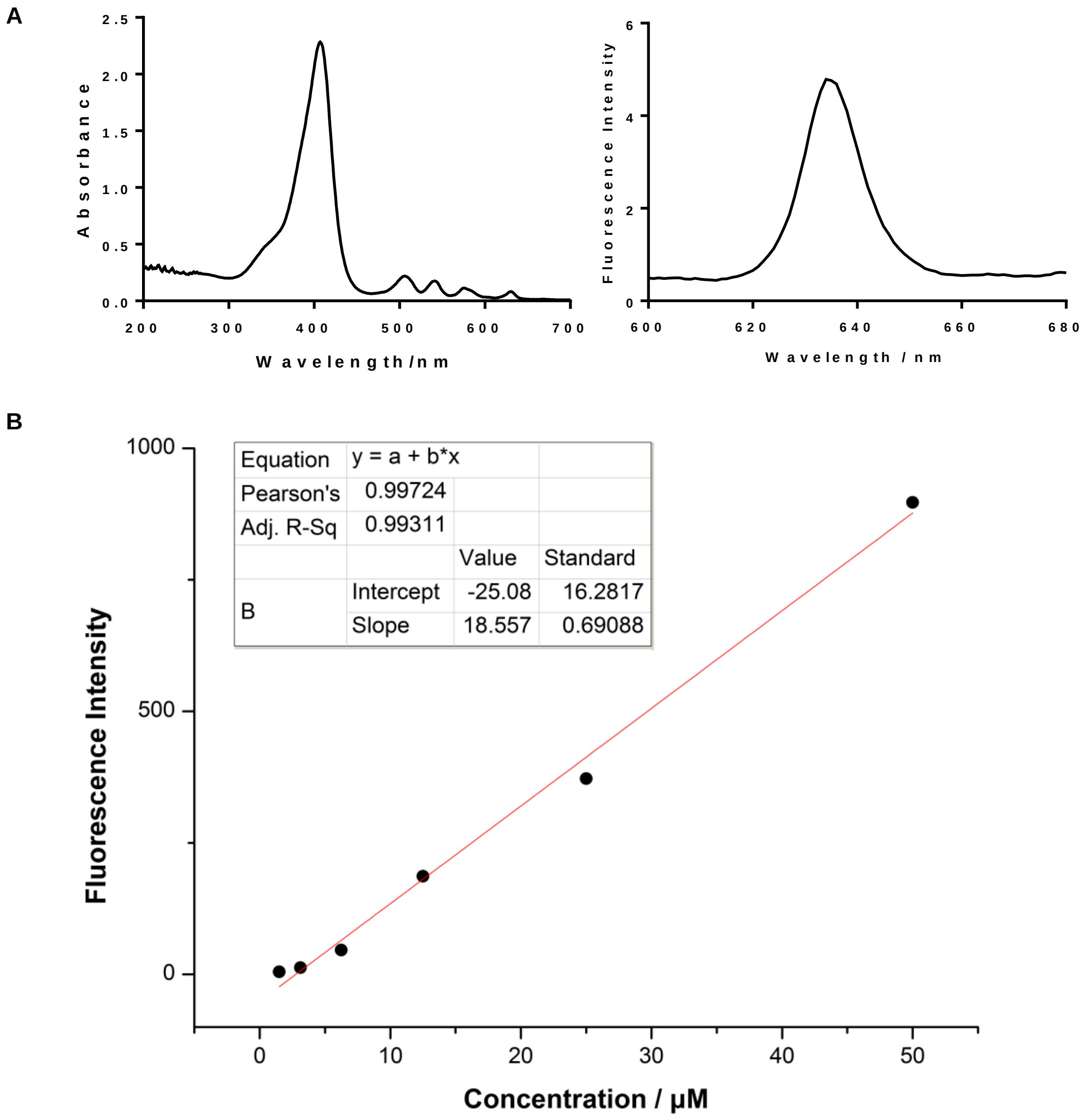

Figure S14. (A) UV-vis absorption spectrum for PPIX and emission spectrum for PPIX-containing supernatent (excited at 505 nm); (B) The plot of fluorescence intensity vs. concentration of PPIX for standard samples.

The concentration of PPIX was measured according to the literature[1]. Hela cells were incubated in the media containing 200 μg/mL CD-ALA-TPP, and the treated Hela cells (about 2×10^5 cells) were homogenized in 4 mL of Tris-HCL (50mmol/L)/ ethanol (9:1), sonicated for 20 min, and then centrifuged for 30 min. The fluorescence of the supernatant was

measured at the excitation wavelength of 505 nm, and fluorescent intensity at 630 nm was recorded. Based on a standard curve in Figure S14B, the concentration of PPIX was calculated as 724 nM/ 10^5 cells.

[1] a) J. A. Stockman, L. S. Weiner, G. E. Simon, M. J. Stuart, F. A. Oski, *J Lab Clin Med* **1975**, *85*; b) F. S. D. Rosa, R. F. V. Lopez, J. A. n. Thomazine, A. n. C. Tedesco, N. Lange, *Pharmaceutical Research* **2004**, *21*, *2247*.

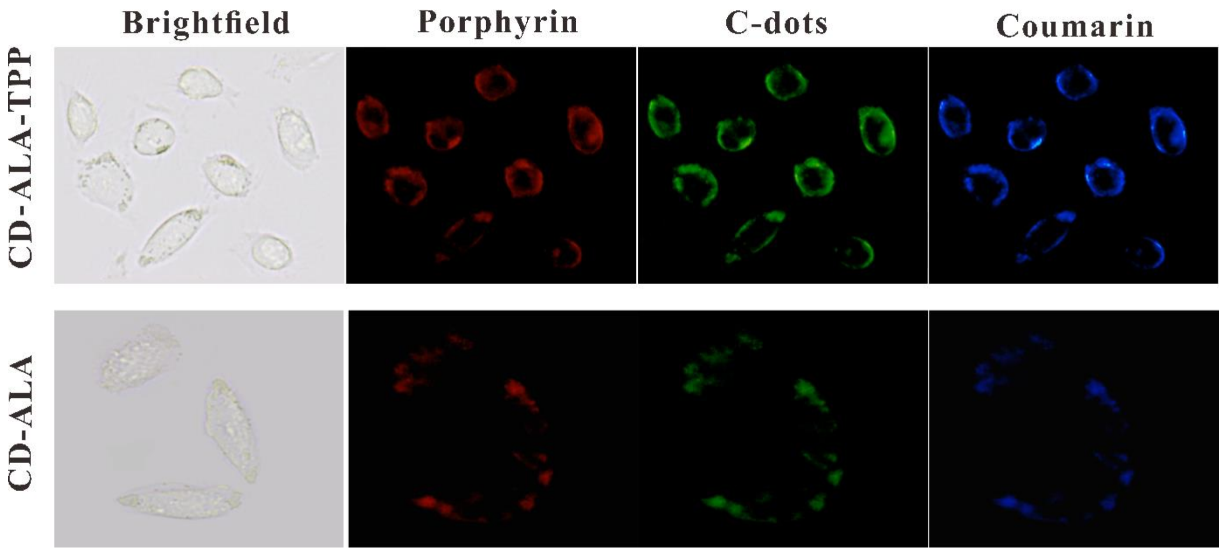

Figure S15. Bright field and fluorescent images for Hela cells treated with 100 μg/mL CD-ALA-TPP or CD-ALA and subject to 30 min of violet (400-450 nm) light irradiation.

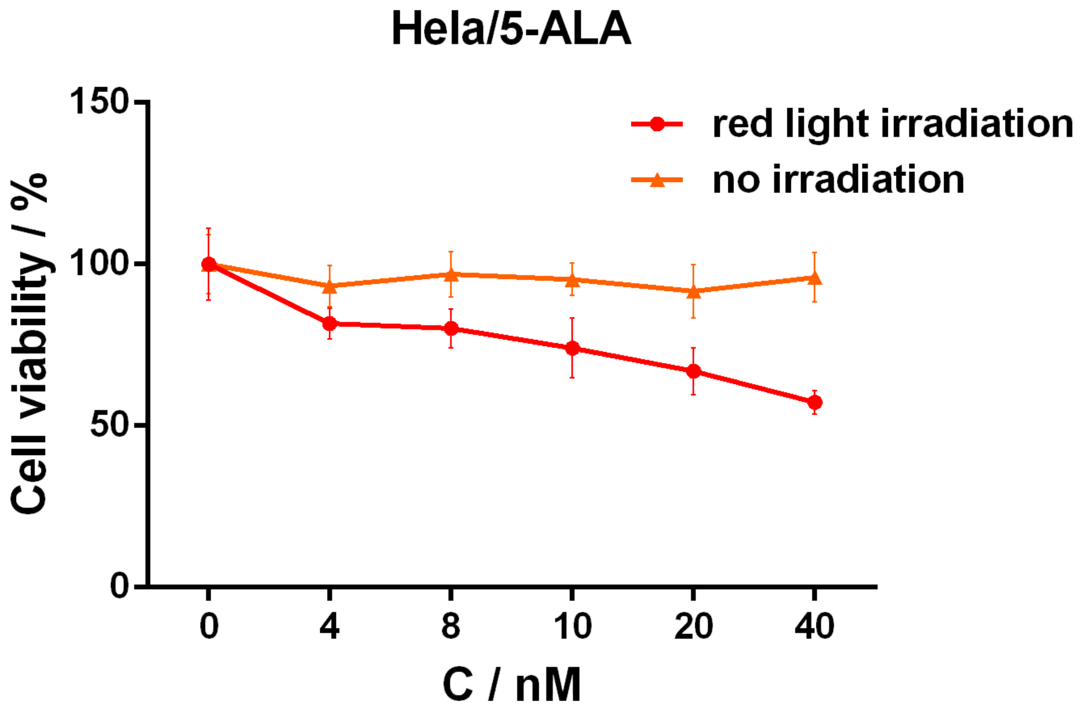

Figure S16. Cells viabilities for Hela cells treated with molecular 5-ALA of varied concentrations. Cell viability was assessed by MTT assay upon 24 h of incubation after being treated with various concentrations of 5-ALA for 4 h (red light or no irradiation). Each concentration was performed independently for three times, and for each independent experiment, the assays were performed in eight samples.

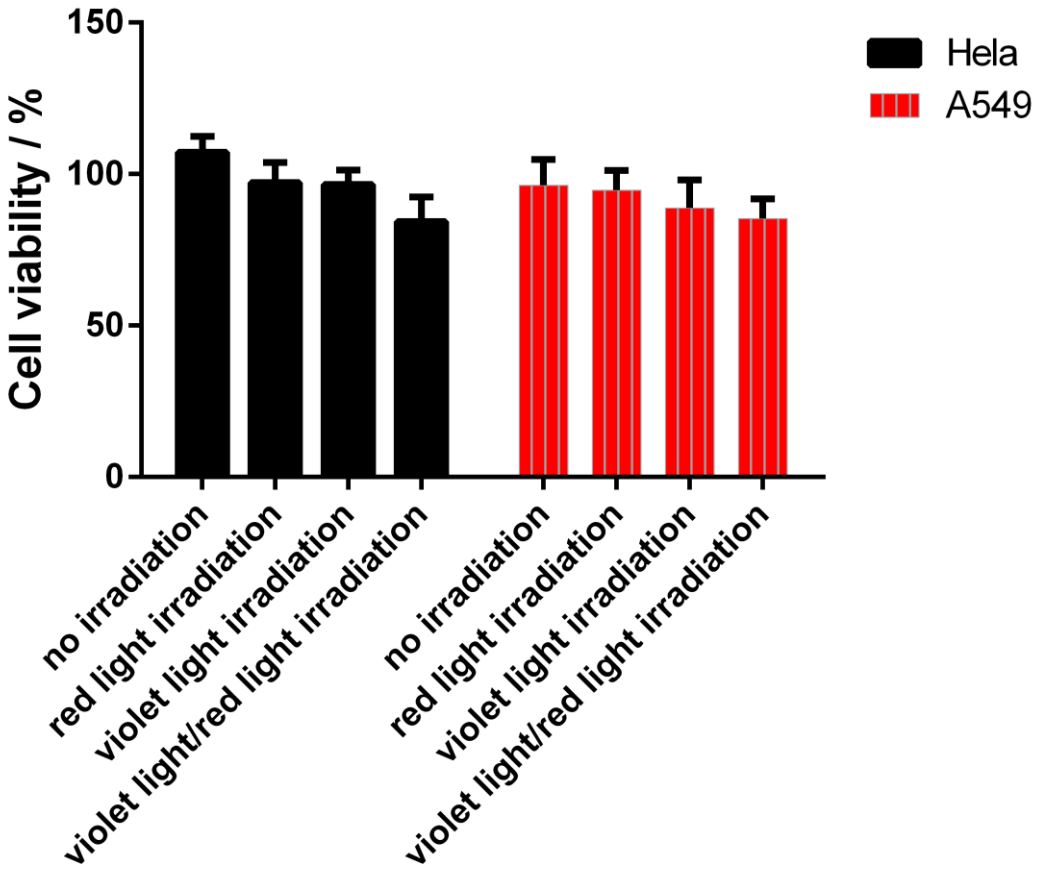

Figure S17. Viability upon different irradiation conditions for Hela and A549 cell lines without being pretreated with the nanosystem.

Cell viability was assessed by MTT assay upon 24 h of incubation after exposure to different irradiation conditions (violet light/red light, violet light, red light or no irradiation). Each condition was performed independently for three times, and for each independent experiment, the assays were performed in eight samples. Data represent mean ± SD from three independent experiments.
